# Exposure to automation explains religious declines

**DOI:** 10.1073/pnas.2304748120

**Published:** 2023-08-14

**Authors:** Joshua Conrad Jackson, Kai Chi Yam, Pok Man Tang, Chris G. Sibley, Adam Waytz

**Affiliations:** ^a^Behavioral Science Department, Booth School of Business, University of Chicago, Chicago, IL 60640; ^b^Management & Organizations Department, National University of Singapore Business School, National University of Singapore, Singapore 117561, Singapore; ^c^Department of Management, Terry College of Business, University of Georgia, Athens, GA 30602; ^d^Department of Psychology, University of Auckland, Auckland, NZ 1142; ^e^Department of Management and Organizations, Kellogg School of Management, Northwestern University, Evanston, IL 60208

**Keywords:** religion, automation, artificial intelligence, cultural evolution

## Abstract

The global decline of religiosity represents one of the most significant societal shifts in recent history. After millennia of near-universal religious identification, the world is experiencing a regionally uneven trend toward secularization. We propose an explanation of this decline, which claims that automation—the development of robots and artificial intelligence (AI)—can partly explain modern religious declines. We build four unique datasets composed of more than 3 million individuals which show that robotics and AI exposure is linked to 21st-century religious declines across nations, metropolitan regions, and individual people. Key results hold controlling for other technological developments (e.g., electricity grid access and telecommunications development), socioeconomic indicators (e.g., wealth, residential mobility, and demographics), and factors implicated in previous theories of religious decline (e.g., individual choice norms). An experiment also supports our hypotheses. Our findings partly explain contemporary trends in religious decline and foreshadow where religiosity may wane in the future.

## Significance

The rise of working robots and artificial intelligence (AI) is changing how humans work and live. Many studies have now examined the economic impact of automation on unemployment, income inequality, and trade. However, far less research has considered the broader cultural implications of robotics and AI. Here, we show that exposure to robots and AI explains religious declines across national cultures, regions within a nation, members of a community, and employees in an organization. These effects hold controlling for wealth, SES, exposure to science, political conservatism, and other technological advances. Our findings suggest that the rise of automation could accelerate secularization throughout the 21st century in many world regions.

Religious decline is accelerating in many world regions. The percent of people identifying as nonreligious has risen more than 10% in nations such as Singapore, Iceland, Chile, and South Korea over the last 10 y (1). The percent of religious “nones” in the United States (US) only rose from 3 to 10% between 1948 and 2005 but then doubled between 2005 and 2020 (2). Why is religion falling in some regions, and why are these declines accelerating in the 21st century? Just as importantly, why is religious decline so uneven—occurring rapidly in some places while others remain highly religious or become even more religious (1)? Religious decline has no single cause, but there may be broad factors that are facilitating this trend, and identifying these factors could explain which groups and individuals are most likely to lose their faith. We suggest that automation is one of these factors and that the rise of automation may explain religious declines across multiple levels of analysis.

Automation refers to robotics and artificial intelligence (AI) technology, which has exponentially advanced in the 21st century. Automation has transformed medicine (3), agriculture (4), meteorology (5), and the service industry (6). AI programs such as ChatGPT and Midjourney show the capacity of AI to generate human language and art. These AI innovations have been especially prominent in nations like Singapore, South Korea, and the US, which have also experienced notable 21st-century religious declines (7). This correlation does not prove any meaningful connection between automation and religious decline, but several lines of research also raise significant reasons why people living and working in automated spaces may become less religious. We especially draw from converging scholarship suggesting that automation may reduce the instrumental value of religion.

In addition to drawing existential and moral value from religion, people use supernatural beliefs for instrumental functions. In many folk religions, shamans use divination rituals to predict weather patterns, fetal sex, and to determine the best cures for illnesses (8, 9). In world religions like Christianity, people pray more and report more subjective faith in God when they fall ill or experience financial hardship (10–12). Technological advancements give people secular alternatives to fulfill these instrumental goals. When people can use technology to predict the weather, diagnose and treat illness, and manufacture resources, they may rely less on religious beliefs and practices for these specific problems (8).

There is a widespread view among scientists of religion that technological developments may reduce the frequency of some supernatural practices but are insufficient to produce wholesale religious decline. Religion remained stubbornly persistent to the encroachment of science and technology during the industrial revolution (13). Ethnographic fieldwork often reports that people retain supernatural beliefs as ultimate explanations of how technology works (14–16). Cognitive science experiments find that Christians believe that religion works in ways that science cannot understand (17, 18). People continue to associate a range of abilities with supernatural agency, even those under the purview of modern science such as curing terminal illnesses (11, 19, 20). These studies have led scholars of religion to reject Max Weber’s rationalist prediction that a rise in science would result in the “disenchantment of the world.”

Automation, however, represents a new frontier of technology with novel characteristics. Here, we suggest that exposure to automation technology (robotics and AI) may encourage religious declines, even above and beyond general exposure to science and other forms of technology. This claim is based on recent research on lay perceptions of automation. Such studies show that people ascribe automation technology with abilities that border on supernatural. For example, people perceive Google as having a unique level of agency shared only by Christians’ perceptions of God (21) and associate robots and AI with gods more than with humans (22). In many domains, people trust algorithms over trained human experts, a phenomenon called “algorithm appreciation” (23). These perceptions are not always merited, as humans often surpass algorithms in predictive abilities. Nevertheless, many people believe that AI allows people to “play god” in a way that previous scientific and technological advances have not, with some commentators suggesting these perceptions will persist as AI becomes increasingly sophisticated (24).

We document evidence of these perceptions in study S1 (*SI Appendix*). This study shows that participating in a day-long seminar on AI led business executives (*n* = 76) to believe that automation allows humans to “break” laws of nature, gives humans “superhuman” abilities, and allows humans to “do things that we have never been able to do before.” We propose that exposure to automation may decrease religiosity because of this perception of human exceptionalism: Historically, people have deferred to supernatural agents and religious professionals to solve instrumental problems beyond the scope of human ability. These problems may seem more solvable for people working and living in highly automated spaces.

These mechanisms underlying automation and religious decline resemble Norris and Inglehart’s existential security model of secularization (25–27), but also contain distinct elements. The existential insecurity model predicts that rising wealth and stability has driven religious decline because people have experienced fewer of the existential concerns that make religion appealing (25). This thesis is supported by research showing that religiosity rises following natural disasters and warfare (28, 29) and falls when countries become wealthier and more prosperous (25). Our automation hypothesis resembles this model because new forms of automation are typically designed to meet human needs and make life easier. But we also emphasize people’s perceptions of whether technology can alleviate their needs and help with goal pursuit. People may perceive AI as having capacities that they do not ascribe to traditional sciences and technologies and that are uniquely likely to displace the instrumental roles of religion. We therefore predict that automation exposure should predict religious declines across nations and people, even controlling for variation in wealth and other forms of technological and scientific exposure.

The primary purpose of this work is to empirically evaluate the link between automation and religious decline, and we do this with four longitudinal studies and one experiment. Our first two studies operationalize automation through international and regional trends in industrial robots. We combine these robotics data with large surveys of religiosity in over 2 million individuals across 68 nations (study 1) and over 1 million individuals across 110 metropolitan areas in the US (study 2) to test whether the prevalence of robots can explain which regions of the world and the US have experienced the greatest 21st-century religious declines. At the individual level, we predict that exposure to automation should predict religious decline above and beyond exposure to other forms of science and technology. Research on religion and science has found that exposure to science often has little effect on personal religiosity (14, 16, 30, 31). We predict that unlike science, people’s exposure to automation negatively predicts future religiosity and could even predict deconversion. In study 3, we track the religiosity of 69,021 people in New Zealand over 11 y to test whether occupational exposure to AI is associated with losing belief in God. In study 4, we test whether occupational exposure to AI can explain declining religiosity across employees of an organization in Indonesia as it integrates AI technology.

Our final study is an experiment that tests whether learning about automation technology temporarily reduces religious conviction more than learning about equally impressive scientific advances. We also probe for the properties of automation that may mediate this effect. We acknowledge that a negative effect of automation on religion could be driven by a heterogeneous assortment of mechanisms, which may operate to different degrees in studies 1 to 4. In study 5, we focus on one of these mechanisms, which is people’s belief that AI to operate outside the laws of nature that constrain human science. In our general discussion and *SI Appendix*, we discuss and empirically test other plausible mechanisms that could explain why religiosity declines in highly automated spaces.

## Results

Data and code are available from https://osf.io/stby4/. All statistical tests are two-tailed.

### Study 1: Robotics Exposure Explains Religious Declines across World Nations.

Our first study tracked religious declines across world nations. We operationalized automation through each nation’s yearly operational stock of industrial robots, defined as an “automatically controlled, reprogrammable multipurpose manipulator programable in three or more axes” by the International Federation of Robots (IFR). We operationalized religiosity through yearly survey data on the proportion of people across nations ( ∑n = 2,014,633) who answered “yes” to the question “Is religion an important part of your daily life?” Variations of this religious importance item are frequently used to measure religiosity across cultures because it can gauge religiosity across a variety of religious traditions and does not make assumptions about religious content (e.g., monotheism). Combining these datasets gave us data on 68 countries from 2006 to 2019, which we used to test whether robotics exposure could explain 21st-century religious decline.

We controlled for several other variables. Our primary control variables involved other forms of technological change: mobile phone subscriptions per capita and the share of population with access to electricity. Measuring telecommunication and energy development allowed us to test the role of robotics above and beyond general technological infrastructure. We also controlled for population size, since operational stock of robots could be larger in more populous countries. We also controlled for GDP per capita and individual choice norms around fertility. We controlled for these latter two variables because the existential theory of secularization suggests that wealth reduces religiosity because it increases certainty and stability (25), whereas other theories focus on value change (32); one view suggests that religious decline occurs when cultures emphasize individual choice norms (e.g., contraception) over profertility norms (27). Our Methods section contains more information about each of these covariates.

**Fig. 1.**
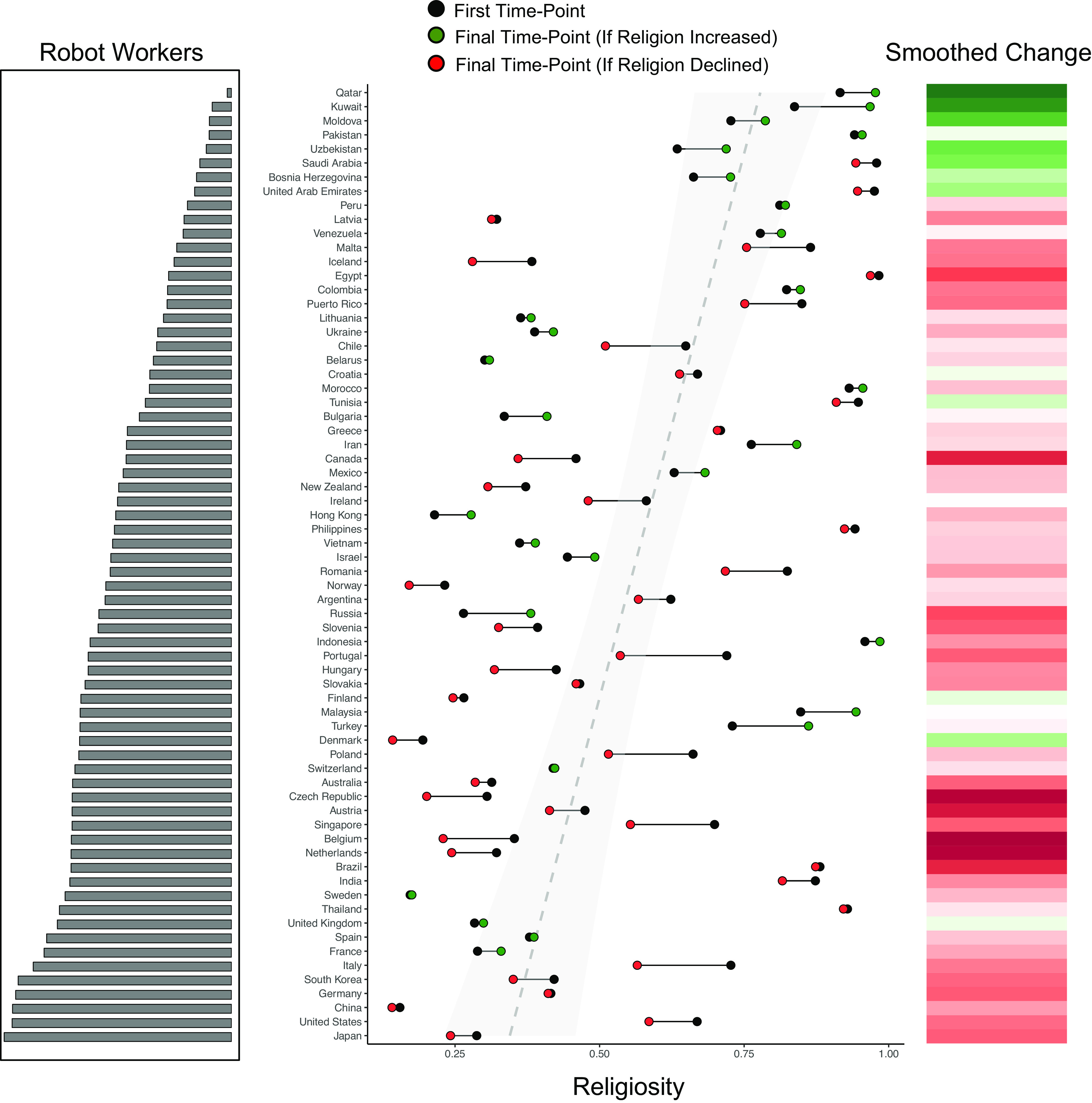
Robotics exposure and global religious decline. Each nation is ordered in terms of robot prevalence, which is displayed as a log-transformed histogram on the left side of the plot. The central panel shows the mean importance of religiosity for each nation at the first timepoint of the dataset (white nodes) and at the final timepoint of the dataset (colored nodes). Green final timepoint nodes represent rising religious importance and red nodes represent declining religious importance. The dashed trendline represents the correlation between mean importance of religion (across all timepoints) and robotics exposure, and the shaded region indicates SE. The gradient bar on the right side of the plot displays the degree of religious change across the sample of countries more prominently using the same color scheme. This gradient has been smoothed so that each bar indicates the mean religious change score of the horizontally adjacent country and its two *y* axis neighbors. The trendline shows that robotics correlates negatively with religion cross-sectionally, and the color gradient shows that high-robotics countries have experienced greater religious decline than low-robotics countries.

Cross-sectional models with intercepts varying randomly across nations found that robotics exposure was robustly and negatively associated with religiosity across the globe (Table 1, model 1). This negative association replicated controlling for GDP per capita and population size (Table 1, model 2) and continued to reach significance controlling for telecommunication and energy development (Table 1, model 3). A longitudinal model with intercepts and slopes randomly varying across nations next estimated whether robotics was linked to declines in religiosity. In this model (Table 1, model 4), the interaction between robotics and year was significant. This effect replicated controlling for the interaction of telecommunication development with year (Table 1, model 5) and energy development with year (Table 1, model 6). Neither energy nor telecommunication development significantly explained declines in religiosity. In the model where we held these covariates constant, nations with a high operational stock (+1 SD) of robots experienced an approximately 3% decline in religiosity per decade (*P* = 0.01), whereas nations with low operational stock (−1 SD) showed approximately a 0.1% increase per decade (*P* = 0.95). This may seem like a small effect, but it was substantially larger than any other geopolitical variable, and these small effects can fuel large divergences in religiosity over time. Fig. 1 illustrates these dynamics.

**Table 1.** Robotics exposure and global religious decline

	Religiosity					
	Estimate (SE)					
	(1)	(2)	(3)	(4)	(5)	(6)
Constant	0.06 (0.12)	0.06 (0.12)	0.04 (0.12)	0.03 (0.12)	0.03 (0.12)	0.02 (0.12)
Robotics exposure	−0.08^***^ (0.02)	−0.09^***^ (0.02)	−0.06^**^ (0.02)	−0.06 (0.03)	−0.06 (0.03)	−0.06 (0.03)
Year				−0.02 (0.02)	−0.03 (0.02)	−0.03 (0.02)
Telecom. development		0.03^**^ (0.01)	0.04^**^ (0.01)	0.03^*^ (0.01)	0.05^*^ (0.02)	0.04^*^ (0.02)
Energy development		−0.04^*^ (0.02)	−0.02 (0.02)	−0.01 (0.03)	−0.005 (0.03)	0.005 (0.03)
GDP per capita			−0.16^*^ (0.07)	−0.07 (0.09)	−0.07 (0.09)	−0.07 (0.09)
Population size			−0.04 (0.12)	0.04 (0.12)	0.05 (0.13)	0.05 (0.13)
Choice norms			−0.60^***^ (0.12)	−0.65^***^ (0.12)	−0.65^***^ (0.12)	−0.65^***^ (0.12)
Robotics exposure x year				−0.02^*^ (0.01)	−0.03^*^ (0.01)	−0.03^*^ (0.01)
Telecom. development x year					0.01 (0.01)	0.01 (0.01)
Energy development x year						0.01 (0.01)
Observations	809	801	594	594	594	594
Log likelihood	92.30	90.49	70.71	95.24	92.16	88.90
Akaike Inf. Crit.	−176.60	−168.97	−123.43	−164.49	−156.31	−147.81
Bayesian Inf. Crit.	−157.81	−140.86	−83.95	−107.46	−94.90	−82.00

*Note.* Estimates are presented outside parentheses, and SE are presented inside parentheses. All estimates have been standardized via z-scoring. Exact *P* values are presented in *SI Appendix*. *P < 0.05; **P < 0.01; ***P < 0.001.

These cross-cultural analyses show that robotics exposure, measured here through the density of industrial robots, can explain variation in religiosity around the world and variation in global religious decline from 2006 to 2019. Robotics exposure was associated with religious decline above and beyond other forms of technological development, such as telecommunications development and energy development. Each of these results held controlling for GDP per capita and population size.

In our supplemental analyses, we show that key findings replicate when controlling for spatial autocorrelation and when removing majority-Muslim countries, which had low rates of robot workers and also low rates of religious decline. Analyses also replicate when we interact all variables with year instead of just technological innovations and when we control for an alternative measure of individualism which is more outdated but includes more nations. One interesting finding that emerges in this supplemental analysis is that individual choice norms around fertility, which have a strong negative cross-sectional relationship with religiosity (Table 1), do not significantly predict 21st-century religious decline. We discuss this curious result more in our general discussion.

### Study 2: Robotics Exposure Explains Religious Declines within a Nation.

Study 2 next tested for the relationship between robotics exposure and religious decline within a single nation—the United States of America (USA). Testing the relationship within the USA offered a more conservative test of our hypothesis, since regions within the USA are more religiously homogenous than world nations and have more similar levels of technological development. We therefore could test whether robotics exposure explains religious decline controlling for other variables, such as income, employment rate, and residential mobility, which do vary considerably across regions of the USA and have been linked to automation (33, 34).

We again measured religiosity through the self-reported importance of religion—measured from 2008 to 2016 across metropolitan areas—and measured robotics exposure through the operational stock of industrial robots. Specifically, we used estimates released from the Brookings Institute of each metropolitan areas’ percent growth in industrial robot operational stock (henceforth called robotics growth) between 2010 and 2015. We combined these metrics into a dataset which also included estimates of median income, unemployment, and residential mobility (reverse coded as number of nonmovers) across metropolitan areas. This dataset contained estimates from 110 metropolitan areas and over 1 million individuals (*Materials and Methods*) within the USA, with time-varying religion data from 2008 to 2016 and time-invariant data on robotics growth, income, unemployment, and residential mobility. See *Materials and Methods* for more information about all variables. *SI Appendix*, Fig. S2 is a map of the metropolitan areas in our analysis organized by religious decline and robotics growth.

Cross-sectional models with intercepts varying randomly across metropolitan areas and states found that metropolitan areas with high rates of robotics growth showed no significant differences in religiosity compared to metropolitan areas with low rates of robotics growth (Table 2, model 1), and this association remained null after controlling for unemployment, median income, population size, and number of nonmovers (Table 2, model 2). This cross-sectional association between robotics growth and religiosity is less meaningful across metropolitan areas than across world nations because the distribution of robot workers in the USA has strong regional constraints based on the availability of warehouse space, unionization density, and proximity to travel networks (35). Our critical test was therefore whether robotics growth would explain religious declines. In this test of change over time—a longitudinal model with random intercepts and slopes—we found that robotics growth interacted with time and significantly explained religious declines (Table 2, model 3). Metropolitan areas with higher levels of robotics growth (+1 SD) experienced an approximately 3% yearly decline in religion each decade (*P* = 0.006)—mirroring the effect size we observed in study 1—while metropolitan areas with lower levels of robotics growth (−1 SD) experienced approximately a 0.5% yearly rise in religion (*P* = 0.67). This effect replicated controlling for the interaction of income and year (Table 2, model 4), population size and year, and number of nonmovers and year (Table 2, model 5). No other factor explained religious decline in these models.

**Table 2.** Robotics growth and religious decline within the united states

	Religiosity				
	Estimate (SE)				
	(1)	(2)	(3)	(4)	(5)
Constant	−0.10 (0.15)	0.002 (0.14)	0.002 (0.14)	0.001 (0.14)	−0.002 (0.14)
Robotics growth	−0.02 (0.05)	0.03 (0.05)	0.02 (0.05)	0.02 (0.05)	0.02 (0.05)
Year			−0.02 (0.01)	−0.02 (0.01)	−0.02^*^ (0.01)
% Unemployed		0.10 (0.08)	0.11 (0.08)	0.11 (0.08)	0.10 (0.08)
Median income		−0.39^***^ (0.08)	−0.39^***^ (0.08)	−0.39^***^ (0.08)	−0.39^***^ (0.08)
Population size		0.24 (0.13)	0.24 (0.13)	0.24 (0.13)	0.24 (0.13)
Nonmovers		−0.13 (0.12)	−0.12 (0.12)	−0.12 (0.12)	−0.12 (0.12)
Robotics growth x year			−0.02^*^ (0.01)	−0.02^*^ (0.01)	−0.02^*^ (0.01)
Median income x year				0.01 (0.01)	0.003 (0.01)
Population size x year					−0.02 (0.03)
Nonmovers x year					0.04 (0.03)
Observations	883	856	856	856	856
Log likelihood	−229.76	−207.11	−210.19	−213.57	−218.11
Akaike Inf. Crit.	469.52	432.22	446.38	455.15	468.23
Bayesian Inf. Crit.	493.44	474.99	508.16	521.68	544.26

*Note.* Estimates are presented outside parentheses, and SE are presented inside parentheses. All estimates have been standardized via z-scoring. % Unemployed is only displayed as a main effect because models failed to converge when % unemployed was interacted with year. Exact *P* values are presented in *SI Appendix*. *P < 0.05; **P < 0.01; ***P < 0.001.

These analyses show that robotics exposure can explain religious declines within a nation as well as across nations. Robotics growth was associated with religious declines across USA metropolitan areas controlling for changes in income, residential mobility, and unemployment. In our *SI Appendix*, we present additional analyses where the timespan of religious change is restricted to the same time window in which we have available data on robotics growth (2010 to 2015).

In sum, studies 1 to 2 showed that robotics exposure explained religious decline across and within nations. However, research on the ecological fallacy (36) and Simpson’s paradox (37) shows that group-level associations sometimes do not replicate, and can even reverse, at the individual level. Studies 1 to 2 also focused on exposure to automation through robotics, but this exposure can also happen through occupational work with AI algorithms. Studies 3 to 4 addressed both of these limitations to show that occupational exposure to AI was associated with declines in religiosity across individuals.

### Study 3: AI Exposure Explains Religious Declines in a Community Sample.

Study 3 was a preregistered analysis of occupational AI exposure and religiosity within an 11-wave longitudinal study, which was conducted between 2009 and 2020 in a community sample. Participants in this multiwave study answered several questions about their personal characteristics and their social attitudes. Nine waves of the study included binary items asking people if they believed in God and if they identified as religious or nonreligious (key religion items were omitted from waves 1 and 6). Participants also free-reported their occupation (if any) in all waves, which was manually coded by research assistants into one of 1,036 categories (e.g., “social security assessor” and “debt collector”). In total, 69,021 individuals participated in at least one wave of the study and 46,680 individuals reported their religion in multiple waves of the study (see *Materials and Methods* and *SI Appendix* for more information about sample and recruiting). Our critical hypothesis was that occupational AI exposure would be associated with lower religiosity across individuals and religious decline within individuals.

We measured occupational AI exposure by incorporating occupation-level metadata from O*Net, a large occupational database which has classified occupations based on the importance of different occupational qualities (see *Materials and Methods* for more information). We used data on the importance of programming as a proxy for AI exposure. This proxy appeared face valid since many of the jobs with high importance of programming also involve AI exposure (e.g., “Software Engineer” and “Web Developer”). We also controlled for importance of biology, chemistry, mathematics, and medicine/dentistry to ensure that generalized scientific exposure or scientific education did not confound AI exposure. We also controlled for SES, age, gender, and political conservatism (38). Our main analyses focused on God belief because it allowed us to test whether AI exposure was associated with religious beliefs rather than just self-reported importance of religiosity (which we had found in studies 1 to 2). We found similar results using the religious identification variable, and we summarize those results in *SI Appendix*. In general, God belief was stable across the study—God belief at wave 1 correlated at 0.75 with God belief at wave 11—but a notable proportion of people (17.39%) changed their belief at least once across waves.

**Fig. 2.**
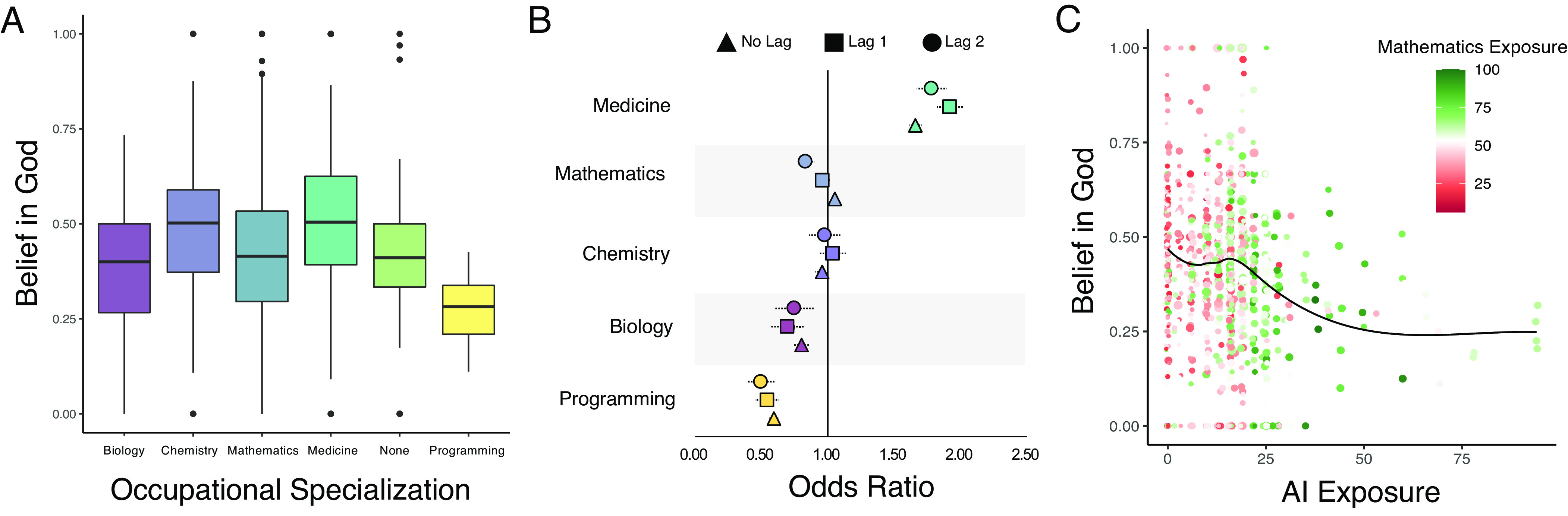
Occupational AI exposure and belief in God. (*A*) A boxplot representing the central tendency and distribution of God belief among workers who worked in occupations with high exposure to biology, chemistry, mathematics, medicine/dentistry, programming/AI, or none of the categories (an importance score of less than 25/100 on all science categories). (*B*) The relationship between exposure to different scientific domains and God belief at no lag, a one-wave lag, and a two-wave lag. Dashed error bars represent 95% CI. (*C*) A scatterplot of God belief on AI exposure. Nodes are occupations, node color represents mathematics exposure, and the trendline is a loess curve.

Baseline analyses of occupational AI exposure and God belief showed that a random slopes and intercepts model outperformed a random intercepts model, $\chi^{2}$ = 34.42, *P* < 0.001. In this random slopes and intercepts model, AI exposure and religiosity were negatively and significantly associated, *b* = −0.59, *SE* = 0.06, *OR* = 0.55, *t* = −9.33, *P* < 0.001, 95% *CI*s [−0.72, −0.47]. Since occupational AI exposure was standardized through z-scoring, the odds ratio suggested that people with jobs that were one SD higher than the mean on occupational exposure to AI were 45% less likely to believe in God compared to people in occupations that had a mean level of exposure to AI. Subsequent models found that this association remained statistically significant controlling for SES, age, gender, and political conservatism (Table 3, model 1), and after controlling for generalized scientific exposure (Table 3, model 2). In subsequent models, we added lagged terms representing occupational exposure to AI at previous timepoints in the survey. In this model, the second-order lag was significantly negatively associated with God belief above and beyond the contemporaneous effect (Table 3, model 3; see *SI Appendix* for models including higher-order lags). Occupational exposure to AI may increase someone’s likelihood of losing their belief in God, even if they subsequently move into an occupation that no longer involves AI exposure. Fig. 2 visualizes these effects.

**Table 3.** AI exposure and god belief in a community sample

	Belief in God		
	Estimate (SE)		
	(1)	(2)	(3)
Constant	−2.18^***^ (0.11)	−2.22^***^ (0.11)	−1.86^***^ (0.24)
Timepoint	−0.22^***^ (0.01)	−0.22^***^ (0.01)	−0.26^***^ (0.02)
Income	−0.10^***^ (0.03)	−0.17^***^ (0.03)	−0.15^*^ (0.07)
Gender	−2.21^***^ (0.08)	−2.10^***^ (0.08)	−2.62^***^ (0.19)
Age	1.19^***^ (0.04)	1.18^***^ (0.04)	1.50^***^ (0.11)
Conservatism	0.97^***^ (0.02)	0.97^***^ (0.02)	0.77^***^ (0.04)
AI exposure	−0.53^***^ (0.04)	−0.52^***^ (0.04)	−0.11 (0.07)
Biology exposure		−0.22^***^ (0.06)	−0.37^**^ (0.13)
Chemistry exposure		−0.05 (0.05)	−0.03 (0.10)
Mathematics exposure		0.05 (0.03)	0.02 (0.06)
Medicine/dentistry exposure		0.51^***^ (0.05)	0.76^***^ (0.10)
AI exposure (lag 1)			−0.26^***^ (0.07)
AI exposure (lag 2)			−0.63^***^ (0.10)
Observations	106,956	106,392	30,305
Log likelihood	−48,817.33	−48,509.15	−12,541.42
Akaike Inf. Crit.	97,654.65	97,046.30	25,114.84
Bayesian Inf. Crit.	97,750.46	97,180.35	25,247.95

*Note.* Estimates are presented outside parentheses, and SE are presented inside parentheses. All occupational exposure variables have been standardized via z-scoring for presentation. Exact *P* values are presented in *SI Appendix*. *P < 0.05; **P < 0.01; ***P < 0.001.

We also fit models which used a within-person centering procedure to separately estimate the within-person and between-person associations between occupational AI exposure and God belief (39). In order for these models to converge, we needed to restrict our sample to individuals who participated in at least six waves of the survey (*n* = 5,542). Results showed that occupational AI exposure was negatively associated with God belief at both the between-person level, *b* = −0.08, *SE* = 0.01, *OR* = 0.93, *t* = −6.74, *P* < 0.001, 95% *CI*s [−0.10, −0.05], and the within-person level, *b* = −0.02, *SE* = 0.008, *OR* = 0.98, *t* = −2.43, *P* = 0.02, 95% *CI*s [−0.03, −0.004]. After controlling for the generalized scientific exposure proxies, these between-person, *b* = −0.07, *SE* = 0.01, *OR* = 0.93, *t* = −6.59, *P* < 0.001, 95% *CI*s [−0.10, −0.05], and within-person, *b* = −0.02, *SE* = 0.008, *OR* = 0.98, *t* = −2.18, *P* = 0.03, 95% *CI*s [−0.03, −0.002], relationships remained negative and statistically significant. In other words, occupational AI exposure explained variation in religious belief across individuals, but also religious decline in the same individual over time.

Our *SI Appendix* contain several supporting analyses. These include a replication of our findings using the religious identification as an outcome instead of God belief, robustness checks which replicate our results using different subgroups of participants to rule out the possibility that our findings were confounded with selective attrition, additional descriptive statistics concerning our key variables, additional models which control for general education rather than specific scientific knowledge, and a discussion of alternative modeling procedures. All analyses support our general conclusion that occupational exposure to AI is associated with lower religiosity between individuals and religious declines within individuals.

### Study 4: AI Exposure Explains Religious Declines within an Organization.

Study 4 was a preregistered three-wave (time-lagged) study in which we measured occupational AI exposure in a manufacturing plant as it integrated AI technology. We followed 238 employees within the organization over time, directly measuring their exposure to AI and their religious profiles (intrinsic religiosity and fundamentalism) at time 1 (T1), their perceived religious importance over the last week and their frequency of religious behaviors over the last week at time 2 (T2), and supervisor-reports of employee workplace behaviors at time 3 (T3). Study 4 was the most conservative test of our hypothesis because it featured the smallest and most homogenous sample (participants were primarily Muslim; see *Materials and Methods* and *SI Appendix*) and took place over the narrowest time frame (3 wk). This study also allowed us to explore the consequences of AI-linked religious decline for workplace behavior.

Because of space limitations, we present the T1 and T2 findings in this paper, which replicate our findings in study 3. Initial analyses found that T1 AI exposure was negatively associated with T2 religiosity, *r*(236) = −0.24, *P* < 0.001. This association was stronger for the items measuring participants’ subjective religious importance over the last week, *r*(236) = −0.27, *P* < 0.001, than for the items measuring frequency of religious behaviors over the last week, *r*(236) = −0.17, *P* < 0.001. This association alone is limited because less religious people may have been faster to adopt AI technology. However, we found that the negative association replicated when we controlled for T1 religious fundamentalism and intrinsic religiosity, *b* = −0.18, *SE* = 0.08, β = −0.19, *t*(229) = −2.17, *P* = 0.03, 95% *CI*s [−0.35, −0.02], suggesting that AI predicts declines in religiosity as well as cross-sectional variation in religiosity.

We provide more analyses and statistics in our *SI Appendix*. These analyses also consider downstream associations with participants’ T3 workplace behaviors that have been linked to religiosity (40–42) (e.g., unethical behavior, trust, incivility, and organizational citizenship behavior, which are face-valid indicators of prosociality). We find that declines in religiosity predict changes in many of these variables.

In sum, studies 3 to 4 showed that exposure to AI is linked to declines in religiosity at the individual level. Occupational exposure to AI correlated with lower levels of religiosity across and within individuals, even controlling for covariates. Moreover, AI exposure predicted future declines in religion.

### Study 5: Learning about AI Decreases Religious Conviction in an Experimental Paradigm.

Our final study was a preregistered experiment testing whether learning about advances in AI would temporarily decrease religious conviction to a greater extent than learning about other scientific advances. This experiment also explored the properties of AI that might explain why learning about AI reduces religious conviction. We were particularly interested in whether people perceive AI, like God, to operate outside the laws of nature compared to traditional sciences such as chemistry, biology, and medicine. To do this, we asked people whether they associated AI and other scientific advances with discovering and applying “laws of nature,” with the implicit view that domains associated with laws of nature like gravity, matter, and motion would be more constrained by these laws. This approach has limitations (e.g., being associated vs. constrained by laws of nature are not interchangeable), but we felt that this approach was less demand-laden than nudging participants to think of automation vs. science as God-like, which could interfere with our central dependent variable, religious conviction.

We used a between-subjects design to test our hypotheses. A general sample of 1,400 participants with a range of religious beliefs (*Materials and Methods*)—were randomly assigned to read about three advances in science or AI, which were matched on domain (e.g., language, medicine, or agriculture) and length (one paragraph). For example, participants in the AI condition read about ChatGPT, whereas participants in the science condition read about a recently published study showing that Broca’s area is involved in the production of sign language. Participants rated each automation/scientific advance on its impressiveness, technological sophistication, and the extent that it was associated with laws of nature. They also rated the extent that each advance increased vs. decreased their religious conviction (see *Materials and Methods* for more details).

We fit general linear models with Gaussian estimation testing whether AI advances decreased religious conviction relative to science advances, controlling for their impressiveness and technological sophistication. AI advances were rated as similarly impressive to science advances, *b* = 0.04, *SE* = 0.06, *t* = 0.66, *P* = 0.51, *95% CIs* [−0.08, 0.17], and more technologically sophisticated than scientific advances, *b* = 1.26, *SE* = 0.07, *t* = 18.27, *P* < 0.001, *95% CIs* [1.13, 1.40]. Our results are virtually identical regardless of these controls (*SI Appendix*).

**Fig. 3.**
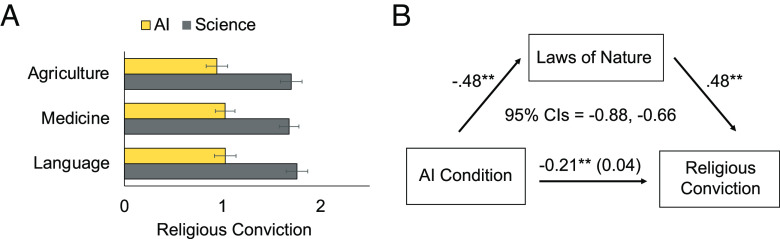
Illustration of study 5 results. (*A*) Mean religious conviction for participants in the AI and science conditions of study 5. (*B*) Estimates from a 5,000-sample bootstrapped mediation model, fit in lavaan for study 5, in which association with laws of nature fully explains why learning about AI reduces religiosity more than learning about science.

As we predicted, participants viewed AI advances as less associated from laws of nature than scientific advances, *b* = −1.56, *SE* = 0.08, *t* = −20.34, *P* < 0.001, *95% CIs* [−1.71, −1.41]. We also found that participants reported less religious conviction in the AI condition vs. the science condition, *b* = −0.71, *SE* = 0.10, *t* = −6.95, *P* < 0.001, *95% CIs* [−0.91, −0.51]. The effect on religious conviction was larger among participants who identified as religious, *b* = −1.05, *SE* = 0.14, *t* = −7.36, *P* < 0.001, *95% CIs* [−1.34, −0.77], than nonreligious, *b* = −0.19, *SE* = 0.10, *t* = −1.85, *P* = 0.06, *95% CIs* [−0.39, 0.01], presumably because religious conviction had a greater range of variance for religious participants. Effects broken down by each domain of innovation are summarized in Fig. 3. Fig. 3 also reports a 5,000-sample bootstrapped mediational model in which find that the association with laws of nature fully mediated the effect of automation on religious conviction.

## Discussion

The expression “Deus ex Machina,” or “God out of the Machine,” describes an improbable event that resolves a literary plotline. The expression is a metaphor; God does not actually appear out of machines in these stories. But the expression’s inverse could be more literal. Over the last several decades, the world has witnessed a kind of “Machina ex Deus”: Innovations in AI and robotics have spread rapidly around the world and captured public interest, whereas religiosity has declined in many regions at a historically unprecedented pace (1). Here, we suggest that these trends are not correlated by coincidence, but that there are meaningful properties of automation which encourage religious decline.

We support our hypothesis with five studies comprising millions of people. Our studies show that religious declines have been fastest in nations (study 1) and geographical regions (study 2) with high levels of robots and that this relationship cannot be explained by key technological or socioeconomic variables. We also found that entering occupations that involve more AI exposure is associated with lower levels of religiosity between individuals and declining belief in God within individuals (study 3) and that exposure to AI is associated with religious decline in an organization incorporating AI technology (study 4). In study 5, we show that learning about advances in AI is associated with greater reductions in religious conviction than learning about scientific advances. Our studies demonstrate that automation is linked to religious decline across multiple religious traditions (e.g., Christian, Muslim, and Buddhist), world regions (e.g., North America, South Asia, and Oceania), and levels of analysis.

Our *SI Appendix* provide robustness tests for each of our studies, and they also summarize additional studies which explore nuances and implications of these findings. Study S1, as noted in the introduction, finds that learning about AI in an intensive day-long seminar increased people’s belief that technology has given humans superhuman abilities (i.e., to “play God” and “break laws of nature”). Attending this seminar also decreased the perceived importance of prayer and service attendance at work among highly religious people, but not less religious people. Studies S2-3 explore how religiosity correlates with favorability toward automation. Religious people around the world and across the United States are less favorable to automation than nonreligious individuals, even controlling for their favorability toward science (study S2), and view automation as less compatible with religion than scientific disciplines (study S3). There are multiple potential explanations behind this negative correlation. For example, favorability toward automation may lead to religious decline, resulting in a negative correlation between the variables. Religious individuals may also report more negative attitudes toward automation because they feel more threatened by automation than nonreligious individuals.

Our introduction focuses on the possibility that automation is decreasing the instrumental value of religion. However, we also acknowledge that there may be other mechanisms at play across our studies, and we empirically explore these mechanisms in our other supplemental studies. For example, one alternative mechanism is that the activities and challenges inherent in occupations involving automation are less likely to inspire religiosity compared to the activities and challenges involved in occupations that do not involve automation. Study S4 finds some support for this mechanism, showing that activities associated with AI occupations are viewed as more focused on mechanistic “how” questions rather than existential “why” questions, and by virtue of this association, they are less likely to inspire religious devotion than activities associated with other fields of science and technology. Finally, study S5 tests whether religious people anticipate becoming less religious when they enter a job in AI vs. in medicine, providing evidence that religious individuals accurately predict the religious declines that we observed in study 3. Altogether, these studies further support our hypothesis, but also foreshadow future research questions (e.g., to what extent do people make a conscious decision to deconvert in automated spaces?).

There are important limitations of this research program. For example, our theory focuses on religion as a way to satisfy instrumental human needs, but people also turn to religion for moral guidance and purpose. Because people are generally averse to machines making decisions in moral contexts (43), religion might endure as a moral institution in the age of automation. This is ultimately an open question, however, given the emergence of AI in moral decision-making (44) and given that some people have begun losing faith in religion’s moral value (particularly in Catholic cultures where church scandals have undermined the moral authority of the church) (32). We encourage future research that integrates these streams of research to identify the distinct causes of religious decline. In our *SI Appendix*, we write more about how to synthesize these different literatures.

Another limitation of our research is that we conceptualized automation broadly. For example, in studies 1 to 2, we measured automation through the size of the robotics industry, whereas in studies 3 to 4, we measured automation through individual people’s workplace exposure to AI. At the national and regional level, it is difficult to disentangle these measures. Nations with large robotics industries also have higher levels of AI integration than nations with smaller robotics industries (7). However, these different kinds of automation exposure could be studied separately at the individual level, and we encourage future research to test how different kinds of automation exposure may have different effects on religious decline. For example, when automation resembles mechanization (e.g., factory assembly lines), people should be less convinced of automation’s potential to impact and improve human life. We encourage future research to test whether these occupations are boundary conditions for our theory.

In early theories of secularization, Marx (45), Weber (46), Durkheim (47), and Freud (48) each wrote that technological advancements inherent in industrialization would contribute to a widespread loss of religion. Contrary to these predictions, industrialization did not spell the end for religion. But automation may bring late vindication for this thesis, at least in industrialized countries. Our findings show that the rise of AI and robotics has been a crucial and overlooked mechanism for explaining religious declines. Our data do not imply that religion is facing worldwide extinction—if anything, religion is polarizing across world regions. But our studies do suggest that current trends in automation may foreshadow religiosity trends in the near and distant future.

## Materials and Methods

Our *SI Appendix* contain additional information about sampling and variable characteristics. All data and code are publicly available at https://osf.io/stby4/. This project page also contains preregistrations for studies 3 to 5 and our supplemental studies.

### Ethics Approval.

Studies 1 to 2 did not involve original human subject data collection. Our original human subject studies were approved by Institutional Review Boards. Study 3 was approved by the University of Auckland’s Human Ethics Committee, with reviews and renewals every 3 y (the most recent reference number is #014889). Study 4 was approved by National Sun Yat-Sen University’s IRB (IRB# 200604). Study 5 was approved by Northwestern University’s IRB (STU# STU00206763).

### Study 1.

#### Industrial robots.

Our estimates of industrial robot operational stock came from the IFR. The IFR defines industrial robots as “automatically controlled, reprogrammable multipurpose manipulators programmable in three or more axes.” The IFR provides yearly estimates of industrial robots installed across all sectors, but also separately provides the number of robots installed in construction, electricity, manufacturing, mining, and agriculture. We log-transformed the operational stock estimates prior to analyses since they showed a strong positive skew.

#### Religiosity.

Our estimates of religiosity came from the Gallup World Poll, which is the most comprehensive longitudinal and global source of data on religion. The Gallup World Poll surveyed 2,014,633 between 2006 and 2020 with the question “is religion an important part of your daily life?” Although this item does not assess specific religious beliefs, it is a widely used measure of religiosity because it applies to people from a variety of religious traditions, and it has been commonly used in cross-cultural studies. Participants answered the item using “yes” or “no,” and the Gallup World Poll publicly published yearly data on the proportion of people in each society who answered “yes.”

#### Technological development.

Our estimates of the share of people with mobile phone subscriptions came from the International Telecommunications Union, which publishes yearly data on the number of mobile phone subscriptions per 100,000 people. Estimates of the share of people with access to electricity came from the World Development Indicators, which is published yearly by the World Bank. See *SI Appendix* for more information. We log-transformed both technological development indicators prior to analyses since they showed a strong positive skew.

#### Control variables.

We operationalized wealth as GDP per capita based on purchasing power parity in 2017 US dollars, which we retrieved for each country-year observation using data from the World Bank. No data were available from Venezuela, and so Venezuela was not included in models controlling for GDP per capita. Estimates of population size came from the United Nations Population Division. We log-transformed both GDP per capita and population size prior to analyses since they showed a strong positive skew. We computed individual choice norms using the same six items as Inglehart (27): whether homosexuality, divorce, and abortion are ever justifiable, whether men have a greater right to a job than women, and whether higher education is more important for boys than girls. We computed two versions of this scale: individual choice norms from countries’ most recent wave of the WVS at the time of our Gallup Data, and the average of countries’ individual choice norms across waves five and six, which overlapped with our Gallup data. The two metrics correlated highly (*r* = 0.99), and the results were identical with either measure. We use the most recent scores here. Fewer data points were available for our analyses including individual choice norms because we could only analyze nations (*n* = 49) which had data available from both the WVS and Gallup.

### Study 2.

#### Robotics Growth.

The Brookings Institute published data—originally gathered by the IFR—on the percent change in industrial robots across American metropolitan areas from 2010 to 2015, using the same definition of industrial robots as our measure in study 1. Increases in industrial robots ranged from 1.75% in Shreveport-Bossier City, Louisiana, to 33.50% in Charleston, West Virginia. Unlike our nation-level measure of industrial robot operational stock, robotics growth was normally distributed across metropolitan areas.

#### Religiosity.

Our estimates of religiosity came from the Gallup “U.S. Dailies” poll, which asks individuals across metropolitan areas “is religion an important part of your life?” As with the World Poll, U.S. Dailies provides their data in terms of an aggregate percent of the people who respond “Yes” to this question. These data were available from 2008 to 2016 and contained approximately 175,000 individuals each year.

#### Control Variables.

Our estimates of median income, unemployment, and residential mobility (nonmovers) came from the 2010 U.S. Census. Each variable was positively skewed, and so we log-transformed all estimates prior to analyses.

### Study 3.

#### Participants.

We drew our sample from the 2009 to 2020 waves of the New Zealand Attitudes and Values Survey (NZAVS). The NZAVS is a longitudinal national study of social attitudes, personality, and health outcomes of New Zealanders. The methodology of the study, including the measures and the sampling procedure, have been extensively described in other publications (49, 50).

#### Religiosity.

In the main text, we focus on God belief, which was measured by the “yes” or “no” response to the question “Do you believe in a God?” In *SI Appendix*, we also analyze data on religious identification, which was measured by the “yes” or “no” response to the question “Do you identify with a religion and/or spiritual group?” The NZAVS has measured other forms of religiosity (e.g., prayer frequency) in select waves, but we focused on items that were measured throughout the course of the study. The NZAVS also measures strength of religious identification within religious individuals, but we did not analyze this item since our focus was on leaving religion.

#### Occupational AI exposure.

We measured occupational AI exposure through the properties of participants’ occupations. Participants in the NZAVS self-reported their occupation, which research assistants then classified into 1,036 different unique categories. We trained two research assistants to match these categories into the 874 workplace codes from O*Net based on the responsibilities of the occupation. For example, “Finance Manager” was matched to “Financial Manager,” and “Child Care Centre Manager” was matched to “Education and Childcare Administrators, Preschool and Daycare.” To ensure that this matching was reliable, the two research assistants completed same 250 occupations and we established that they were translating the occupations at a sufficiently reliable rate (Krippendorf’s alpha = 0.81). Research assistants then divided the remaining occupations and worked separately on matching them. After each NZAVS occupation had been matched to an O*Net code, we downloaded O*Net data on “cross-functional skills” and focused on “Importance of Programming” as a proxy for an occupation in computer science that would involve high occupational exposure to AI.

### Study 4.

#### Participants.

We invited 250 employees of a food processing manufacturer in Indonesia to participate in our study. Upon receiving the consents, the company administrative team conducted a short briefing with these employees. All surveys were completed in form of paper-and-pencil questionnaires on the last day of the work week and participants in the assembly hall of the company. In total, 238 employees (136 men, 102 women; *M*_age_ = 33.40, *SD*_age_ = 8.42; 9 Christian, 191 Muslim, 4 Jain, 6 Ba’hai, 1 Taoist, 5 Nonreligious, and 22 “Other”) completed all waves, including 36 managers. Participants completed the study across three survey waves, which each occurred a week apart. This time-lagged design is common for capturing dynamics in organizations over time (51, 52).

#### Occupational AI exposure (T1).

We surveyed exposure to AI through a three-item measure in which participants used a 1 to 5 scale to answer how frequently in the last week they had a) initiated work-related interaction with AI, b) interacted with AI at work, and c) interacted with AI informally at work. Participants were given a definition of AI alongside the items as “systems or software equipped with autonomous learning, problem-solving, and decision-making capabilities, where it can learn from externally acquired data and use learning to achieve specific goals.”

#### Intrinsic religiosity (T1).

Participants completed a short-form of the intrinsic religiosity questionnaire, which was adapted by Schneider, Kriefer, & Bayraktar (53) from Allport & Ross (54). The measure contained eight items, including “I enjoy reading about my religion” and “I try hard to live all my life according to my religious beliefs.” Participants answered the items on a one (Strongly agree) – five (Strongly disagree) scale. We reverse-coded the scale so that higher values meant more intrinsic religiosity.

#### Religiosity (T2).

We measured religiosity using a composite scale that we developed for this study, with three items tapping perceived religious importance and three items tapping frequency of religious behaviors. Participants rated the importance of a) God/Allah/gods, b) prayer, and c) their religious community from one (Not at All Important) – five (Very Important). We encouraged participants to focus on their religious “thoughts and activities over the last week” to make the scale more contextually sensitive. Participants rated the frequency of three religious behaviors (attending religious services, reading religious scripture, engaging in religious prayer) over the last week using a one (Not at all or less than once) – five (Many times) scale. We fit a varimax-rotated maximum likelihood factor analysis to determine that the scale was best captured with a one-factor solution.

#### Workplace behaviors (T3).

We adapted widely used measures of organizational citizenship behavior, goal progress, counterproductive workplace behavior, incivility, unethical behavior, task proficiency, and trust. We measured each variable using a supervisor report (i.e., each employees’ behaviors were rated by their immediate supervisor). For the sake of space, we summarize each measure in greater depth and provide sources in *SI Appendix*.

### Study 5.

#### Participants.

Our total sample in study 5 was 1,371 participants (674 men, 687 women, and 9 “Other”; *M*_age_ = 44.85, *SD*_age_ = 12.53). This sample combined two studies: A pilot study (*n* = 394), and a preregistered replication of the pilot (*n* = 977) with distinct samples. The results are identical if we analyze only the main study (*SI Appendix*), so we elected to present results with the largest sample size possible. This was a general sample that we did not filter based on religion. In total, 573 participants identified as Atheist (*n* = 166), agnostic (*n* = 233), or as “none” (174) when they reported their religious identity. The most common religious identity was Christian (*n* = 341), followed by Catholic (*n* = 283). All participants provided informed consent before participating.

#### Manipulation.

Participants were randomly assigned to the AI (*n* = 687) or science (*n* = 684) condition. Participants in both conditions read about three recent advances. These advances were matched across conditions to focus on language (Chat GPT in the AI condition; a study of Broca’s area and sign language in the science condition), medicine (AI-generated X-rays in the AI condition, a study showing the association between Vitamin D and skin cancer in the science condition), and agriculture (IoT in the AI condition, an explanation of photosynthesis in the science condition). The advances were described in one paragraph each. We sourced the paragraphs mostly from press releases, and we provide them in *SI Appendix*.

#### Measures.

Participants responded to the items 1) “This is an example of discovering laws of nature,” 2) “This is an example of applying laws of nature,” 3) “This is impressive,” 4) “This is technologically sophisticated,” 5) “Reading this strengthens my religious conviction,” 6) “Reading this makes me feel closer to God.” Participants rated these items using a one (“Strongly Disagree”) – seven (“Strongly Agree”) scale for each domain, and we collapsed across domains in our analyses because results were highly similar for each domain. Items 1 to 2 indicated association with (vs. dissociation from) laws of nature, items 3 to 4 were preregistered control variables, and items 5 to 6 indicated religious conviction.

## Supplementary Material

Appendix 01 (PDF)

## Data Availability

Multiple data have been deposited in Open Science Framework (https://osf.io/stby4/) (55).
