# Supplementary material for: Exposure to automation explains religious declines
Source: Proc Natl Acad Sci U S A. 2023 Aug 14;120(34):e2304748120. doi: 10.1073/pnas.2304748120 (PMC10450436; doi:10.1073/pnas.2304748120)
Supplement: Supplementary file 1 — Appendix 01 (PDF) [file pnas.2304748120.sapp.pdf]

Supplemental Information

1. SUPPLEMENTAL THEORETICAL INFORMATION 2
Cultural Evolutionary Theories of Religion and Religious Decline 2
Contribution of Our Work 3
2. SUPPLEMENTAL INFORMATION FOR STUDY 1 3
List of Countries by Religiosity and AI Exposure in Study 1 4
More Information about Variables in Study 1 5
Results by Type of Robot in Study 1 6
Exact p-Values of Models in Table 1 9
Additional Visualizations for Study 1 10
Additional Robustness Checks for Study 1 11
3. SUPPLEMENTAL INFORMATION FOR STUDY 2 13
List of Metropolitan Areas by Religiosity and AI Exposure 14
Exact p-Values of Models in Table 2 16
Visualizing Robotics Growth and Religious Decline Across Metropolitan Areas 16
AI Interest and Religiosity Interest in the United States Over Time 17
4. SUPPLEMENTAL INFORMATION FOR STUDY 3 19
Descriptive Statistics for Study 3 Variables 19
Incorporating Third-Order Lagged Terms in Study 3 19
Exact p-Values in Table 3 20
Replicating Study 3 Results with Religious Identification Instead of God Belief 20
Additional Robustness Tests for Study 3 21
Ruling out Alternative Modeling Strategies in the Study 3 Dataset 23
5. SUPPLEMENTAL INFORMATION FOR STUDY 4 23
Study 4 Workplace Behavior Measures 23
Study 4 Full Multiple Regression Models 24
Study 4 Supplemental Measures 25
6. SUPPLEMENTAL INFORMATION FOR STUDY 5 27
Stimuli in Study 5 27
Robustness Analyses for Study 5 29
7. STUDY S1 29
8. STUDY S2A-B 31
9. STUDY S3 33
10. STUDY S4 34
11. STUDY S5 35
REFERENCES 37

1. Supplemental Theoretical Information

Religious decline is a major area of study across the human sciences. We view our research as most relevant to research on religious decline from a cultural evolutionary perspective. Here we expand on this theorizing and situate our research within this broader space.

Cultural Evolutionary Theories of Religion and Religious Decline

There are many models of cultural evolution, but we subscribe to a dual inheritance model in which human behavior and belief systems arises jointly from (a) genetic information, which individuals inherit from their parents via reproduction, and (b) cultural information, which individuals inherit from their society via social learning (1). Therefore, humans might be religious either because they have genetically inherited psychological profiles that make them prone to belief in supernatural agents, or because they have socially learned these beliefs from their parents, peers, and other members of their society.

Findings on the genetic transmission of religion remain mixed (2). Some research has proposed that inherited psychological traits such as intuitive thinking style (3,4), theory of mind (5), and death anxiety (6) predispose individuals to religion. However, large studies have failed to support several of these patterns (7–9), and these bio-psychological mechanisms are poorly suited for explaining cross-cultural variation in religion given their focus on mechanisms shared by all humans. Most comparative studies of religious decline have therefore focused on cultural transmission via social learning.

One class of social learning mechanisms involves the *context* in which people learn about religion, including the person from whom they learn about religion. For example, people are more likely to maintain belief into adulthood if they learn about religion from highly devoted caregivers who regularly signal religious commitment through fasting, attending services, and donating to their religious community (7,10,11). These signals are alternatively described as “credibility enhancing displays” (CREDs) or “honest signals” because they would be extremely costly if people did not authentically hold their beliefs and they therefore provide an honest cue to religious devotion (12). Childhood exposure to CREDs can explain variation in religiosity both inside and outside the United States and can even predict deconversion age among people who grow up in religious households (10). Declining CREDs are therefore important component of religious decline.

A second class of social learning mechanisms focuses on the *content* of religious beliefs. Early content-based hypotheses focused on qualities that made supernatural agents easy to remember and discuss (13–15). For example, the minimally counterintuitive (MCI) hypothesis suggested that people would be more likely to remember gods who selectively violated lay expectations about physics (e.g., by walking on water), biology (e.g., by gaining immortality), or psychology (e.g., by reading minds) than gods who violated none of these lay expectations or who violated all of them (14). Despite widespread interest in the MCI hypothesis, studies supporting the hypothesis have been critiqued because they confound minimally counterintuitive violations with emotionality (e.g., gods possess traits which are emotionally galvanizing), fitness relevance (e.g., gods possess traits that are relevant to survival), and existential relevance (e.g., gods possess traits that threaten or prolong the lives of believers) (16). A more general critique of the MCI hypothesis is that it only explains which agents people *remember;* it does not explain why people become devoted to gods and spirits. This critique is called the “Mickey Mouse” problem because the MCI hypothesis is equally well suited for explaining the evolution of non-worshipped agents like Mickey Mouse and Santa Claus as it is for explaining the evolution of worshipped agents like Jesus or Allah (16). The related “Zeus” problem is that the MCI hypothesis cannot explain why some gods are worshipped in a particular time and place (e.g., the Christian God) whereas others cease to be worshipped (e.g., Zeus) (17).

More recent analyses of content-biased transmission have tried to directly address the Zeus and Mickey Mouse problems. For example, Swan and Halberstadt (18) directly compared characteristics of active gods and other fictional agents (e.g., Mickey Mouse, Zeus), finding that active gods were ascribed more superhuman powers—especially helpful psychological powers—compared to other fictional agents (Mickey Mouse may be unusual, but he does not have special psychological capacities and will not solve

your problems). Purzycki and McNamara (19) have argued that gods are usually associated with specific community functions that make them deserving of worship and ritual, such as resource management and social norm regulation. Epley and colleagues (20) made a similar claim by showing that people project their own communal concerns onto gods more than other people (see also Purzycki (21)).

These findings are useful because they show how specific features of gods—namely, the possession of superhuman powers and willingness to use these powers to help resolve human problems—are essential for encouraging demonstrations of religious commitment. In other words, most people perceive religion to have an instrumental function. When people appeal to supernatural agents to help them solve problems, these appeals in turn act as CREDs that inspire the next generation of religious adherents. Content-based dual inheritance models of religious evolution are therefore compatible with context-based models.

Contribution of Our Work

We propose that people's religious worship does not only depend on how they perceive gods and religious role-models; it also depends on how people perceive other means of problem solving. If people believe that automation can address needs that they usually depend on religion to address, they may be less likely to seek out supernatural help through petitionary prayer or ritual participation. In turn, this decline in the frequency of religious displays may lead to broader religious declines. People do not need to explicitly compare automation and religion within this dynamic—which might be highly threatening for a religious individual. Rather, religion may simply be salient to people who have no secular means of solving their problems, and these situations may be rarer for people who have access to automation technology.

Our hypothesis is supported by three well-established premises. The first premise is that people perceive automation and religion as sharing similar features and abilities. For example, people see Google as having uniquely high agency, which is shared only by Christians' perceptions of God (22), and implicitly and explicitly associate robots and AI with gods more than humans (23). Our own studies (Study 5, Study S1) support this idea further by showing that people view automation as operating outside laws of nature, and feel that humans can "break" laws of nature after attending a seminar in AI (Study S1). The evidence in our research and past studies suggests that people think, at least implicitly (23), that automation can fulfill many of the needs that they have previously entrusted to supernatural agents.

The second premise is that people are most likely to engage in religious displays when in need of supernatural aid. For example, prayer and ritual participation increase during natural disasters (24,25) and warfare (26,27), situations in which human science has a limited capacity to help people. Conversely, rises in wealth and stability—which reduce dependence on supernatural aid—have foreshadowed declines in religion in the throughout the 20th century (28,29).

The third and final premise is that declines in religious displays can lead to loss of faith among observers. This hypothesis is well-supported by the CREDs literature (11). Studies of CREDs mostly focus on intergenerational transmission, but declining participation in religious displays may also affect the beliefs of people's peers, or people's own beliefs. Multiple lines of research in social psychology suggest that people use behavioral displays to gauge their own beliefs (30,31). If people pray and attend religious services less frequently, this should have a negative effect on their own beliefs.

As noted throughout the main text, we view automation as only one mechanism of religious decline. Other complementary mechanisms are also plausible. For example, whereas our work focuses on people's perceived need for religion, other research has focused on people's perception of religious institutions. Scandals involving the Catholic Church have served as credibility "undermining" displays (CRUDs) which have turned people away their religious communities and churches (32,33). These value-based perspectives complement our needs-based perspective, which focuses more on the perceived instrumental benefits of religion.

2. Supplemental Information for Study 1

List of Countries by Religiosity and AI Exposure in Study 1

Table S1.
List of Countries in Study 1 by Religiosity and AI Exposure

Country	Years Available	Logged AI Exposure	Religiosity
Argentina	14	7.04	0.62
Australia	13	8.86	0.31
Austria	13	8.88	0.47
Belarus	14	4.35	0.35
Belgium	13	8.93	0.33
Bosnia and Herzegovina	12	1.95	0.73
Brazil	14	8.94	0.88
Bulgaria	12	5.13	0.39
Canada	14	5.87	0.41
Chile	14	4.17	0.64
China	7	12.21	0.15
Columbia	14	3.56	0.85
Croatia	12	4.55	0.64
Czech Republic	12	8.88	0.24
Denmark	14	8.47	0.18
Egypt	11	3.50	0.98
Finland	12	8.39	0.27
France	14	10.44	0.29
Germany	14	12.03	0.40
Greece	13	5.81	0.72
Hong Kong	10	6.45	0.25
Hungary	13	7.99	0.35
Iceland	7	3.20	0.33
India	14	9.01	0.82
Indonesia	14	7.88	0.98
Iran	11	5.86	0.84
Ireland	13	6.35	0.53
Israel	14	6.73	0.48
Italy	14	11.05	0.65
Japan	13	12.67	0.25
Kuwait	4	1.07	0.93
Latvia	13	2.64	0.34
Lithuania	13	3.79	0.40
Malaysia	12	8.43	0.91
Malta	11	3.05	0.82
Mexico	14	6.04	0.64
Moldova	14	1.23	0.73
Morocco	4	4.58	0.95
Netherlands	13	8.94	0.30
New Zealand	13	6.29	0.32
Norway	9	7.01	0.20
Pakistan	14	1.24	0.94
Peru	14	2.45	0.82
Philippines	14	6.53	0.94
Poland	14	8.51	0.64
Portugal	13	7.99	0.62
Puerto Rico	2	3.59	0.80
Qatar	3	0.23	0.95
Republic of Korea	13	11.89	0.41
Romania	13	6.76	0.79
Russian Federation	14	7.39	0.32

Saudi Arabia	4	1.76	0.97
Singapore	13	8.88	0.62
Slovakia	11	8.16	0.46
Slovenia	12	7.43	0.39
Spain	14	10.30	0.39
Sweden	14	9.27	0.18
Switzerland	9	8.73	0.40
Thailand	14	9.59	0.95
Tunisia	11	4.80	0.92
Turkey	13	8.44	0.82
Ukraine	14	4.11	0.44
United Arab Emirates	3	2.05	0.94
United Kingdom	14	9.70	0.29
United States	14	12.23	0.64
Uzbekistan	13	1.41	0.58
Venezuela	14	2.69	0.77
Vietnam	9	6.63	0.34

Note. The AI exposure and religiosity variables are averaged across all available years for each country. The exact years available for each country—and the values for those years—can be found in our Study 1 cleaned dataset on https://osf.io/stby4/.

More Information about Variables in Study 1

Share of Population with Electricity Access. Data on access to electricity are collected among different sources: mostly data from nationally representative household surveys (including national censuses) were used. Survey sources include Demographic and Health Surveys (DHS) and Living Standards Measurement Surveys (LSMS), Multi-Indicator Cluster Surveys (MICS), the World Health Survey (WHS), other nationally developed and implemented surveys, and various government agencies (for example, ministries of energy and utilities). Given the low frequency and the regional distribution of some surveys, several countries have gaps in available data. To develop the historical evolution and starting point of electrification rates, a simple modeling approach was adopted to fill in the missing data points - around 1990, around 2000, and around 2010. Therefore, a country can have a continuum of zero to three data points. There are 42 countries with zero data point and the weighted regional average was used as an estimate for electrification in each of the data periods. 170 countries have between one and three data points and missing data are estimated by using a model with region, country, and time variables. The model keeps the original observation if data is available for any of the time periods. This modeling approach allowed the estimation of electrification rates for 212 countries over these three time periods (Indicated as "Estimate"). Notation "Assumption" refers to the assumption of universal access in countries classified as developed by the United Nations. Data begins from the year in which the first survey data is available for each country.

Share of Population with Mobile Phone Access. Mobile cellular telephone subscriptions are subscriptions to a public mobile telephone service that provide access to the PSTN using cellular technology. The indicator includes (and is split into) the number of postpaid subscriptions, and the number of active prepaid accounts (i.e., that have been used during the last three months). The indicator applies to all mobile cellular subscriptions that offer voice communications. It excludes subscriptions via data cards or USB modems, subscriptions to public mobile data services, private trunked mobile radio, telepoint, radio paging and telemetry services.

Discrepancies between global and national figures may arise when countries use a different definition than the one used by the International Telecommunications Union (ITU). For example, some countries do not include the number of ISDN channels when calculating the number of fixed telephone lines. Discrepancies may also arise in cases where the end of a fiscal year differs from that used by ITU, which is the end of December of every year. A number of countries have fiscal years that end in March or June of every year. Data are usually not adjusted for discrepancies in the definition, reference year or the break in comparability in between years are noted in a data note. Missing values are estimated by ITU.

Results by Type of Robot in Study 1

Study 1 measured log-transformed estimates of industrial robot operational stock from the International Federation of Robots (IFR). The IFR provides yearly estimates of industrial robots installed across all sectors, but also provides the number of robots installed in construction, electricity, manufacturing, mining, and agriculture. In our main text analyses, we focused on overall stock, but here we break down the operational stock based on each sector. These analyses, presented in Tables S2-6 show the same significant findings as our main text across a variety of sectors. We find that the results are similar, and statistically significant, across sectors.

Table S2.
Study 1 Analyses with Robots in Agriculture

	Religiosity					
	Estimate (SE)					
	(1)	(2)	(3)	(4)	(5)	(6)
Constant	0.06<br>(0.12)	0.06<br>(0.12)	0.04<br>(0.12)	0.03<br>(0.12)	0.03<br>(0.12)	0.03<br>(0.12)
Industrial Robots in Agriculture	-0.09***<br>(0.02)	-0.10***<br>(0.02)	-0.07***<br>(0.02)	0.01<br>(0.03)	0.01<br>(0.03)	0.01<br>(0.03)
Year				-0.03<br>(0.02)	-0.04*<br>(0.02)	-0.04*<br>(0.02)
Telecom. Development		0.02*<br>(0.01)	0.03**<br>(0.01)	0.03*<br>(0.01)	0.04<br>(0.02)	0.04<br>(0.02)
Energy Development		-0.04*<br>(0.02)	-0.03<br>(0.02)	-0.01<br>(0.03)	-0.01<br>(0.03)	-0.001<br>(0.03)
GDP per Capita			-0.13<br>(0.07)	-0.12<br>(0.09)	-0.12<br>(0.09)	-0.12<br>(0.09)
Population Size			-0.04<br>(0.12)	-0.01<br>(0.12)	-0.003<br>(0.12)	0.004<br>(0.13)
Choice Norms			-0.61***<br>(0.12)	-0.65***<br>(0.12)	-0.65***<br>(0.12)	-0.65***<br>(0.12)
Industrial Robots in Agriculture x Year				-0.03**<br>(0.01)	-0.03**<br>(0.01)	-0.03**<br>(0.01)
Telecom. Development x Year					0.01<br>(0.01)	0.01<br>(0.01)
Energy Development x Year						0.01<br>(0.01)
Observations	809	801	594	594	594	594
Log Likelihood	98.14	95.86	73.25	96.22	93.01	89.70
Akaike Inf. Crit.	-188.27	-179.72	-128.49	-166.43	-158.01	-149.41
Bayesian Inf. Crit.	-169.49	-151.60	-89.01	-109.40	-96.60	-83.60

Note. Estimates are presented outside parentheses, and standard errors are presented inside parentheses. All estimates have been standardized. * $p < .05$; ** $p < .01$; *** $p < .001$.

Table S3.
Study 1 Analyses with Robots in Construction

	Religiosity

	Estimate (SE)					
	(1)	(2)	(3)	(4)	(5)	(6)
Constant	0.06<br>(0.12)	0.06<br>(0.12)	0.03<br>(0.12)	0.04<br>(0.12)	0.03<br>(0.12)	0.03<br>(0.12)
Industrial Robots in Construction	-0.07***<br>(0.02)	-0.09***<br>(0.02)	-0.06**<br>(0.02)	-0.002<br>(0.03)	-0.003<br>(0.03)	-0.01<br>(0.03)
Year				-0.03<br>(0.02)	-0.03<br>(0.02)	-0.03<br>(0.02)
Telecom. Development		0.02*<br>(0.01)	0.04**<br>(0.01)	0.03<br>(0.01)	0.04<br>(0.02)	0.04<br>(0.02)
Energy Development		-0.05*<br>(0.02)	-0.03<br>(0.02)	-0.01<br>(0.03)	-0.01<br>(0.03)	0.002<br>(0.03)
GDP per Capita			-0.15*<br>(0.07)	-0.10<br>(0.09)	-0.11<br>(0.09)	-0.11<br>(0.09)
Population Size			-0.04<br>(0.12)	-0.01<br>(0.12)	-0.004<br>(0.12)	0.003<br>(0.13)
Choice Norms			-0.60***<br>(0.12)	-0.66***<br>(0.12)	-0.66***<br>(0.12)	-0.66***<br>(0.12)
Industrial Robots in Construction x Year				-0.03*<br>(0.01)	-0.03*<br>(0.01)	-0.03**<br>(0.01)
Telecom. Development x Year					0.01<br>(0.01)	0.01<br>(0.01)
Energy Development x Year						0.01<br>(0.01)
Observations	809	801	594	594	594	594
Log Likelihood	92.82	92.50	71.08	95.53	92.24	88.99
Akaike Inf. Crit.	-177.64	-172.99	-124.16	-165.06	-156.48	-147.98
Bayesian Inf. Crit.	-158.86	-144.88	-84.68	-108.03	-95.06	-82.18

Note. Estimates are presented outside parentheses, and standard errors are presented inside parentheses. All estimates have been standardized here so that effect sizes can be compared. * $p < .05$; ** $p < .01$; *** $p < .001$.

Table S4.
Study 1 Analyses with Robots in Electricity

	Religiosity					
	Estimate (SE)					
	(1)	(2)	(3)	(4)	(5)	(6)
Constant	0.06<br>(0.12)	0.06<br>(0.12)	0.04<br>(0.12)	0.03<br>(0.12)	0.03<br>(0.12)	0.03<br>(0.12)
Industrial Robots in Electricity	-0.04***<br>(0.01)	-0.05***<br>(0.01)	-0.03<br>(0.01)	0.03<br>(0.02)	0.03<br>(0.02)	0.03<br>(0.02)
Year				-0.04*<br>(0.02)	-0.04*<br>(0.02)	-0.04*<br>(0.02)
Telecom. Development		0.01<br>(0.01)	0.03**<br>(0.01)	0.03*<br>(0.01)	0.05*<br>(0.02)	0.05*<br>(0.02)
Energy Development		-0.04*<br>(0.02)	-0.02<br>(0.02)	-0.01<br>(0.03)	-0.01<br>(0.03)	0.002<br>(0.03)

	(1)	(2)	(3)	(4)	(5)	(6)
GDP per Capita			-0.19**	-0.13	-0.13	-0.13
			(0.07)	(0.09)	(0.09)	(0.09)
Population Size			-0.09	-0.03	-0.02	-0.02
			(0.12)	(0.13)	(0.13)	(0.13)
Choice Norms			-0.61***	-0.67***	-0.67***	-0.67***
			(0.12)	(0.12)	(0.12)	(0.12)
Industrial Robots in Electricity x Year				-0.02*	-0.02*	-0.02*
				(0.01)	(0.01)	(0.01)
Telecom. Development x Year					0.01	0.01
					(0.01)	(0.01)
Energy Development x Year						0.01
						(0.01)
Observations	809	801	594	594	594	594
Log Likelihood	87.50	83.79	67.31	94.09	90.78	87.44
Akaike Inf. Crit.	-167.00	-155.58	-116.61	-162.18	-153.55	-144.88
Bayesian Inf. Crit.	-148.22	-127.46	-77.13	-105.15	-92.13	-79.08

Note. Estimates are presented outside parentheses, and standard errors are presented inside parentheses. All estimates have been standardized here so that effect sizes can be compared. * *p* < .05; ** *p* < .01; *** *p* < .001.

Table S5.
Study 1 Analyses with Robots in Manufacturing

	Religiosity					
	Estimate (SE)					
	(1)	(2)	(3)	(4)	(5)	(6)
Constant	0.06	0.05	0.03	0.03	0.03	0.03
	(0.12)	(0.12)	(0.12)	(0.12)	(0.12)	(0.12)
Industrial Robots in Manufacturing	-0.07***	-0.10***	-0.06**	-0.04	-0.04	-0.04
	(0.02)	(0.02)	(0.02)	(0.03)	(0.03)	(0.03)
Year				-0.02	-0.03	-0.03
				(0.02)	(0.02)	(0.02)
Telecom. Development		0.03**	0.04***	0.03	0.05*	0.04*
		(0.01)	(0.01)	(0.01)	(0.02)	(0.02)
Energy Development		-0.04*	-0.02	-0.01	-0.003	0.01
		(0.02)	(0.02)	(0.03)	(0.03)	(0.03)
GDP per Capita			-0.16*	-0.07	-0.08	-0.08
			(0.07)	(0.09)	(0.09)	(0.09)
Population Size			-0.03	0.03	0.04	0.05
			(0.12)	(0.13)	(0.13)	(0.13)
Choice Norms			-0.60***	-0.65***	-0.65***	-0.65***
			(0.12)	(0.12)	(0.12)	(0.12)
Industrial Robots in Manufacturing x Year				-0.03*	-0.03*	-0.03**
				(0.01)	(0.01)	(0.01)
Telecom. Development x Year					0.01	0.01
					(0.01)	(0.01)
Energy Development x Year						0.01
						(0.01)

Observations	809	801	594	594	594	594
Log Likelihood	92.30	92.50	70.31	95.28	92.45	89.27
Akaike Inf. Crit.	-176.60	-172.99	-122.61	-164.57	-156.89	-148.54
Bayesian Inf. Crit.	-157.81	-144.88	-83.13	-107.54	-95.48	-82.74

Note. Estimates are presented outside parentheses, and standard errors are presented inside parentheses. All estimates have been standardized here so that effect sizes can be compared. * $p < .05$; ** $p < .01$; *** $p < .001$.

Table S6.
Study 1 Analyses with Robots in Mining

	Religiosity					
	Estimate (SE)					
	(1)	(2)	(3)	(4)	(5)	(6)
Constant	0.06 (0.12)	0.06 (0.12)	0.04 (0.12)	0.04 (0.12)	0.03 (0.12)	0.03 (0.12)
Industrial Robots in Mining	-0.04*** (0.01)	-0.04*** (0.01)	-0.02 (0.01)	0.02 (0.02)	0.02 (0.02)	0.02 (0.02)
Year				-0.04* (0.02)	-0.04* (0.02)	-0.04* (0.02)
Telecom. Development		0.02 (0.01)	0.03** (0.01)	0.03* (0.01)	0.05* (0.02)	0.04 (0.02)
Energy Development		-0.05* (0.02)	-0.03 (0.02)	-0.01 (0.03)	-0.01 (0.03)	-0.001 (0.03)
GDP per Capita			-0.19** (0.07)	-0.11 (0.09)	-0.11 (0.09)	-0.11 (0.09)
Population Size			-0.09 (0.12)	-0.02 (0.12)	-0.01 (0.12)	-0.01 (0.13)
Choice Norms			-0.60*** (0.12)	-0.67*** (0.12)	-0.67*** (0.12)	-0.68*** (0.12)
Industrial Robots in Mining x Year				-0.02* (0.01)	-0.02* (0.01)	-0.02* (0.01)
Telecom. Development x Year					0.01 (0.01)	0.01 (0.01)
Energy Development x Year						0.01 (0.01)
Observations	809	801	594	594	594	594
Log Likelihood	86.99	83.79	67.49	94.34	91.01	87.62
Akaike Inf. Crit.	-165.99	-155.58	-116.98	-162.67	-154.02	-145.24
Bayesian Inf. Crit.	-147.21	-127.47	-77.50	-105.64	-92.60	-79.44

Note. Estimates are presented outside parentheses, and standard errors are presented inside parentheses. All estimates have been standardized here so that effect sizes can be compared. * $p < .05$; ** $p < .01$; *** $p < .001$.

Exact p-Values of Models in Table 1

Table 1 in the main text presents the results of Study 1 with standardized beta values, standard errors, and stars connoting statistical significance. Table S7 presents the same results from Table 1 but with exact *p*-values rather than stars connoting significance.

Table S7.
AI and Global Religious Decline in Study 1 with Exact *p*-values

	Religiosity					
	Estimate (*p* values)					
	(1)	(2)	(3)	(4)	(5)	(6)
Constant	0.06 (p = .65)	0.06 (p = .65)	0.04 (p = .75)	0.03 (p = .79)	0.03 (p = .83)	0.02 (p = .83)
Robotics Exposure	-0.08*** (p < .001)	-0.09*** (p < .001)	-0.06** (p = .003)	-0.06 (p = .08)	-0.06 (p = .08)	-0.06 (p = .08)
Year				-0.02 (p = .19)	-0.03 (p = .12)	-0.03 (p = .12)
Telecom. Development		0.03** (p = .009)	0.04** (p = .001)	0.03* (p = .04)	0.05* (p = .04)	0.04* (p = .05)
Energy Development		-0.04* (p = .04)	-0.02 (p = .24)	-0.01 (p = .83)	-0.005 (p = .87)	0.005 (p = .88)
GDP per Capita			-0.16* (p = .02)	-0.07 (p = .45)	-0.07 (p = .44)	-0.07 (p = .44)
Population Size			-0.04 (p = .71)	0.04 (p = .75)	0.05 (p = .71)	0.05 (p = .67)
Choice Norms			-0.60*** (p < .001)	-0.65*** (p < .001)	-0.65*** (p < .001)	-0.65*** (p < .001)
Robotics Exposure x Year				-0.02* (p = .04)	-0.03* (p = .03)	-0.03* (p = .03)
Telecom. Development x Year					0.01 (p = .32)	0.01 (p = .48)
Energy Development x Year						0.01 (p = .45)
Observations	809	801	594	594	594	594
Log Likelihood	92.30	90.49	70.71	95.24	92.16	88.90
Akaike Inf. Crit.	-176.60	-168.97	-123.43	-164.49	-156.31	-147.81
Bayesian Inf. Crit.	-157.81	-140.86	-83.95	-107.46	-94.90	-82.00

Note. Estimates are presented outside parentheses, and exact *p* values are presented inside parentheses. All estimates have been standardized here so that effect sizes can be compared. * $p < .05$; ** $p < .01$; *** $p < .001$.

Additional Visualizations for Study 1

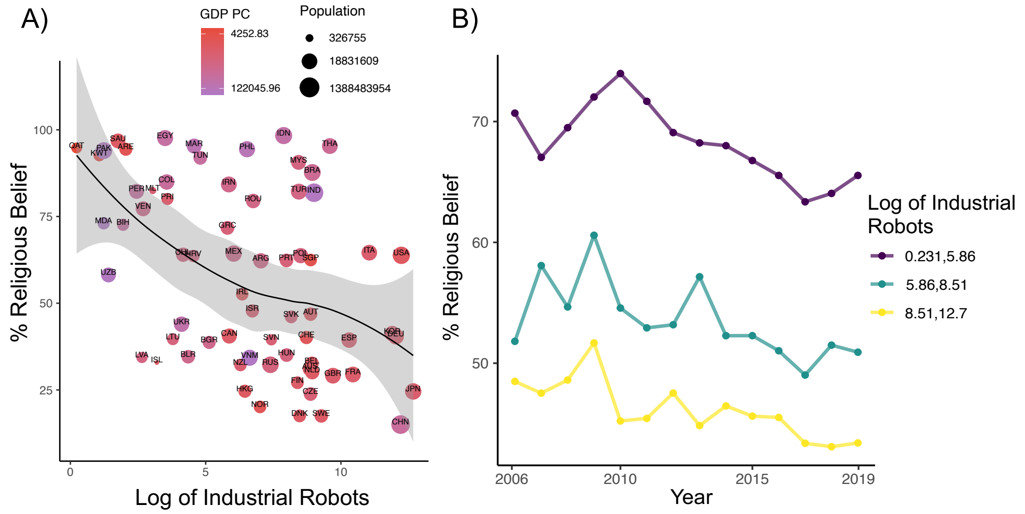

Figure S1. Robotics and Global Religious Decline. Panel A) The cross-sectional association between the operational stock of industrial robots and religious conviction. Nodes represent nations, node size represents population size, and node color represents GDP per capita. Panel B) Yearly religious decline across nations by bottom third, middle third, and top third of industrial robot stock. Line and node color indicate industrial robot stock.

Additional Robustness Checks for Study 1

Table S8 displays two additional Study 1 models which include more robustness checks. Column 1 displays a model in which we interact year with all control variables. Column 2 further includes fixed effects for continents, which controls for spatial autocorrelation, a common source of Type 1 error in cross-cultural surveys. The cross-sectional and longitudinal effects of robotics exposure reach significance in both models. No other factor interacts with time to predict religious decline in these models; choice norms was associated with general levels of religiosity, but not change in religiosity.

Table S8.
Study 1 Robustness Tests

	Religiosity	
	Estimate (SE)	
	(1)	(2)
Constant	0.03 (0.12)	-0.18 (0.25)
Automation	-0.07^{*} (0.04)	-0.07 (0.04)
Year	-0.03 (0.02)	-0.03 (0.02)
GDP per Capita	0.04 (0.02)	0.04 (0.02)
Telecommunications Development	-0.001 (0.03)	-0.01 (0.03)
Energy Development	-0.06 (0.10)	0.02 (0.10)
Population Size	0.04 (0.13)	0.02 (0.12)
Choice Norms	-0.65^{***} (0.13)	-0.73^{***} (0.18)
North America		0.51 (0.61)
South America		1.10^{*} (0.44)
Oceania		-0.06 (0.65)
Europe		0.10 (0.37)

Africa		0.81 (0.47)
Automation x Year	-0.04* (0.02)	-0.04* (0.02)
Telecommunications Development x Year	0.01 (0.01)	0.01 (0.01)
Energy Development x Year	0.01 (0.01)	0.01 (0.01)
GDP Per Capita x Year	0.01 (0.02)	0.01 (0.02)
Population Size x Year	0.02 (0.02)	0.02 (0.02)
Choice Norms x Year	0.004 (0.02)	0.003 (0.02)
Observations	594	594
Log Likelihood	80.23	85.68
Akaike Inf. Crit.	-124.46	-125.36
Bayesian Inf. Crit.	-45.50	-24.46

Note. Estimates are presented outside parentheses, and standard errors are presented inside parentheses. * $p < .05$; ** $p < .01$; *** $p < .001$.

Table S9 reproduces Table 1 without majority Muslim nations, which we defined as nations in which at least 50% of the religiously identified population were Muslim. All key main effects and interactions replicated.

Table S9.
Prevalence of Robot Workers and Global Religious Decline Excluding Muslim Countries

	Religiosity					
	Estimate (SE)					
	(1)	(2)	(3)	(4)	(5)	(6)
Constant	-0.03 (0.13)	-0.03 (0.13)	-0.08 (0.15)	-0.11 (0.16)	-0.12 (0.16)	-0.12 (0.16)
Industrial Robots	-0.09*** (0.02)	-0.10*** (0.02)	-0.07** (0.02)	-0.08* (0.04)	-0.08* (0.04)	-0.09* (0.04)
Year				-0.03 (0.02)	-0.03 (0.02)	-0.04 (0.02)
Telecommunications Development		0.03* (0.01)	0.04** (0.02)	0.04* (0.02)	0.05 (0.03)	0.05* (0.03)
Energy Development		-0.04* (0.02)	-0.03 (0.02)	-0.01 (0.03)	-0.01 (0.03)	0.02 (0.03)
GDP per Capita			-0.19* (0.08)	-0.05 (0.11)	-0.06 (0.11)	-0.06 (0.11)
Population Size			0.01 (0.16)	0.18 (0.17)	0.19 (0.17)	0.20 (0.17)
Choice Norms			-0.32 (0.16)	-0.37* (0.16)	-0.35* (0.16)	-0.36* (0.16)
Industrial Robots x Year				-0.03* (0.02)	-0.03* (0.02)	-0.03* (0.02)
Telecommunications Development x Year					0.01 (0.01)	-0.0004 (0.01)
Energy Development x Year						0.03 (0.02)
Observations	692	690	490	490	490	490

Log Likelihood	24.13	19.18	9.51	39.59	36.36	34.84
Akaike Inf. Crit.	-40.25	-26.37	-1.02	-53.18	-44.72	-39.68
Bayesian Inf. Crit.	-22.10	0.85	36.73	1.35	14.01	23.24

Note. Estimates are presented outside parentheses, and standard errors are presented inside parentheses. * $p < .05$; ** $p < .01$; *** $p < .001$.

Table S10 reproduces Table 1 with an alternative measure of individualism: Hofstede's measure of individualism which was available for 64 of the 68 countries in our sample, whereas Inglehart's measure of individual choice norms was available for only 49. Nevertheless, the results are substantively identical with either measure, and we present the choice norms measure in the main text because it is more up to date; Hofstede's measure was based on data collected in the 1980s.

Table S10.
Prevalence of Robot Workers and Global Religious Decline with Hofstede Individualism

	Religiosity					
	Estimate (SE)					
	(1)	(2)	(3)	(4)	(5)	(6)
Constant	0.06 (0.12)	0.06 (0.12)	0.05 (0.11)	0.05 (0.11)	0.05 (0.11)	0.05 (0.11)
Industrial Robots	-0.08*** (0.02)	-0.09*** (0.02)	-0.08*** (0.02)	-0.08* (0.03)	-0.08* (0.03)	-0.08* (0.03)
Year				-0.04** (0.01)	-0.04** (0.02)	-0.05*** (0.02)
Telecommunications Development		0.03** (0.01)	0.05*** (0.01)	0.05*** (0.01)	0.04* (0.02)	0.05* (0.02)
Energy Development		-0.04* (0.02)	-0.03 (0.02)	-0.01 (0.03)	-0.01 (0.03)	0.04 (0.04)
GDP per Capita			-0.20*** (0.06)	-0.05 (0.08)	-0.05 (0.08)	-0.04 (0.08)
Population Size			-0.05 (0.10)	0.16 (0.10)	0.15 (0.10)	0.17 (0.10)
Hofstede Individualism			-0.47*** (0.11)	-0.56*** (0.11)	-0.56*** (0.11)	-0.57*** (0.11)
Industrial Robots x Year				-0.03* (0.01)	-0.02* (0.01)	-0.02* (0.01)
Telecommunications Development x Year					-0.005 (0.01)	-0.01 (0.01)
Energy Development x Year						0.04* (0.02)
Observations	809	801	750	750	750	750
Log Likelihood	92.30	90.49	99.74	146.36	142.90	142.34
Akaike Inf. Crit.	-176.60	-168.97	-181.47	-266.73	-257.81	-254.68
Bayesian Inf. Crit.	-157.81	-140.86	-139.89	-206.67	-193.12	-185.38

Note. Estimates are presented outside parentheses, and standard errors are presented inside parentheses. * $p < .05$; ** $p < .01$; *** $p < .001$.

3. Supplemental Information for Study 2

List of Metropolitan Areas by Religiosity and AI Exposure

Table S11.
List of Metropolitan Areas in Study 2 by Religiosity and AI Exposure

Metropolitan Area	Years Available	Robotics Growth	Religiosity
Albany-Schenectady-Troy, NY	9	0.30	0.52
Albuquerque, NM	9	0.13	0.63
Anchorage, AK	8	0.19	0.53
Atlanta-Sandy Springs-Roswell, GA	9	0.20	0.74
Augusta-Richmond County, GA-SC	9	0.17	0.79
Austin-Round Rock, TX	9	0.19	0.61
Bakersfield, CA	9	0.21	0.69
Baltimore-Columbia-Towson, MD	9	0.14	0.63
Baton Rouge, LA	9	0.18	0.80
Beaumont-Port Arthur, TX	5	0.20	0.82
Birmingham-Hoover, AL	9	0.26	0.84
Boise City, ID	9	0.20	0.60
Boston-Cambridge-Newton, MA-NH	9	0.18	0.48
Buffalo-Cheektowaga-Niagara Falls, NY	9	0.18	0.60
Burlington-South Burlington, VT	5	0.16	0.41
Cedar Rapids, IA	5	0.17	0.59
Charleston-North Charleston, SC	9	0.17	0.71
Charleston, WV	5	0.34	0.72
Charlotte-Concord-Gastonia, NC-SC	9	0.20	0.75
Chattanooga, TN-GA	9	0.24	0.79
Cincinnati, OH-KY-IN	9	0.21	0.66
Cleveland-Elyria, OH	9	0.17	0.63
Colorado Springs, CO	9	0.16	0.61
Columbia, SC	9	0.21	0.77
Columbus, OH	9	0.15	0.62
Dallas-Fort Worth-Arlington, TX	9	0.18	0.73
Davenport-Moline-Rock Island, IA-IL	5	0.22	0.62
Dayton, OH	9	0.20	0.68
Denver-Aurora-Lakewood, CO	9	0.19	0.55
Des Moines-West Des Moines, IA	9	0.20	0.64
Detroit-Warren-Dearborn, MI	9	0.21	0.64
Duluth, MN-WI	5	0.22	0.60
El Paso, TX	8	0.13	0.73
Erie, PA	5	0.18	0.63
Eugene, OR	9	0.12	0.48
Evansville, IN-KY	5	0.19	0.73
Flint, MI	5	0.14	0.66
Fort Smith, AR-OK	5	0.12	0.77
Fort Wayne, IN	8	0.19	0.71
Fresno, CA	9	0.17	0.68
Grand Rapids-Wyoming, MI	9	0.23	0.68
Green Bay, WI	5	0.21	0.63
Greenville-Anderson-Mauldin, SC	9	0.22	0.80
Harrisburg-Carlisle, PA	9	0.20	0.66
Hartford-West Hartford-East Hartford, CT	9	0.16	0.54
Houston-The Woodlands-Sugar Land, TX	9	0.19	0.73
Huntsville, AL	7	0.13	0.78
Indianapolis-Carmel-Anderson, IN	9	0.16	0.69

Jacksonville, FL	9	0.20	0.69
Kansas City, MO-KS	9	0.20	0.67
Knoxville, TN	9	0.17	0.77
Lansing-East Lansing, MI	6	0.15	0.56
Las Vegas-Henderson-Paradise, NV	9	0.23	0.57
Lexington-Fayette, KY	6	0.15	0.70
Lincoln, NE	5	0.17	0.62
Los Angeles-Long Beach-Anaheim, CA	9	0.16	0.59
Madison, WI	9	0.20	0.48
Medford, OR	5	0.18	0.55
Memphis, TN-MS-AR	9	0.20	0.80
Miami-Fort Lauderdale-West Palm Beach, FL	18	0.18	0.62
Milwaukee-Waukesha-West Allis, WI	9	0.19	0.64
Minneapolis-St. Paul-Bloomington, MN-WI	9	0.19	0.59
Mobile, AL	5	0.29	0.82
Montgomery, AL	5	0.25	0.85
Naples-Immokalee-Marco Island, FL	5	0.27	0.64
New Orleans-Metairie, LA	9	0.21	0.72
New York-Newark-Jersey City, NY-NJ-PA	9	0.17	0.59
Oklahoma City, OK	9	0.20	0.73
Omaha-Council Bluffs, NE-IA	9	0.19	0.65
Orlando-Kissimmee-Sanford, FL	9	0.18	0.66
Peoria, IL	5	0.27	0.67
Philadelphia-Camden-Wilmington, PA-NJ-DE-MD	9	0.14	0.60
Phoenix-Mesa-Scottsdale, AZ	9	0.19	0.60
Pittsburgh, PA	9	0.20	0.66
Portland-South Portland, ME	9	0.15	0.45
Portland-Vancouver-Hillsboro, OR-WA	9	0.21	0.50
Providence-Warwick, RI-MA	9	0.19	0.53
Raleigh, NC	9	0.26	0.67
Reno, NV	5	0.20	0.50
Richmond, VA	9	0.20	0.71
Roanoke, VA	5	0.20	0.72
Rochester, NY	9	0.16	0.55
Rockford, IL	5	0.25	0.62
Sacramento--Roseville--Arden-Arcade, CA	9	0.18	0.54
Salt Lake City, UT	9	0.19	0.60
San Antonio-New Braunfels, TX	9	0.18	0.71
San Diego-Carlsbad, CA	9	0.20	0.56
San Francisco-Oakland-Hayward, CA	9	0.26	0.45
Scranton--Wilkes-Barre--Hazleton, PA	9	0.15	0.65
Seattle-Tacoma-Bellevue, WA	9	0.21	0.48
Shreveport-Bossier City, LA	6	0.02	0.83
South Bend-Mishawaka, IN-MI	5	0.09	0.66
Spokane-Spokane Valley, WA	9	0.20	0.59
Springfield, MA	9	0.13	0.51
Springfield, MO	7	0.22	0.72
St. Louis, MO-IL	9	0.21	0.66
Syracuse, NY	9	0.08	0.55
Tallahassee, FL	5	0.11	0.69
Tampa-St. Petersburg-Clearwater, FL	9	0.20	0.63
Toledo, OH	9	0.28	0.63
Topeka, KS	5	0.18	0.65

Tucson, AZ	9	0.18	0.59
Tulsa, OK	9	0.19	0.74
Urban Honolulu, HI	9	0.21	0.56
Utica-Rome, NY	5	0.13	0.58
Virginia Beach-Norfolk-Newport News, VA-NC	9	0.19	0.69
Washington-Arlington-Alexandria, DC-VA-MD-WV	9	0.16	0.62
Wichita, KS	9	0.23	0.71
Wilmington, NC	5	0.13	0.71
Youngstown-Warren-Boardman, OH-PA	9	0.16	0.69

Exact p-Values of Models in Table 2

Table S12.
Robotics Growth and Religious Decline in the United States in Study 2 with Exact *p*-Values

	Religiosity				
	Estimate (95% CIs)				
	(1)	(2)	(3)	(4)	(5)
Constant	-0.10 ($p < .001$)	0.002 ($p < .001$)	0.002 ($p < .001$)	0.001 ($p < .001$)	-0.002 ($p < .001$)
Robotics Growth	-0.02 ($p = .73$)	0.03 ($p = .53$)	0.02 ($p = .61$)	0.02 ($p = .61$)	0.02 ($p = .60$)
Year			-0.02 ($p = .07$)	-0.02 ($p = .46$)	-0.02* ($p = .48$)
% Unemployed		0.10 ($p = .21$)	0.11 ($p = .20$)	0.11 ($p = .20$)	0.10 ($p = .20$)
Median Income		-0.39*** ($p < .001$)	-0.39*** ($p < .001$)	-0.39*** ($p < .001$)	-0.39*** ($p < .001$)
Population Size		0.24 ($p = .06$)	0.24 ($p = .06$)	0.24 ($p = .06$)	0.24 ($p = .06$)
Non-Movers		-0.13 ($p = .27$)	-0.12 ($p = .31$)	-0.12 ($p = .31$)	-0.12 ($p = .31$)
Robotics Growth x Year			-0.02* ($p = .03$)	-0.02* ($p = .02$)	-0.02* ($p = .03$)
Median Income x Year				0.01 ($p = .35$)	0.003 ($p = .75$)
Population Size x Year					-0.02 ($p = .37$)
Non-Movers x Year					0.04 ($p = .13$)
Observations	883	856	856	856	856
Log Likelihood	-229.76	-207.11	-210.19	-213.57	-218.11
Akaike Inf. Crit.	469.52	432.22	446.38	455.15	468.23
Bayesian Inf. Crit.	493.44	474.99	508.16	521.68	544.26

Note. Estimates are presented outside parentheses, and standard errors are presented inside parentheses. All estimates have been standardized for presentation, so that effect sizes can be compared. % Unemployed is only displayed as a main effect because models failed to converge when % unemployed was interacted with year. * $p < .05$; ** $p < .01$; *** $p < .001$.

Visualizing Robotics Growth and Religious Decline Across Metropolitan Areas

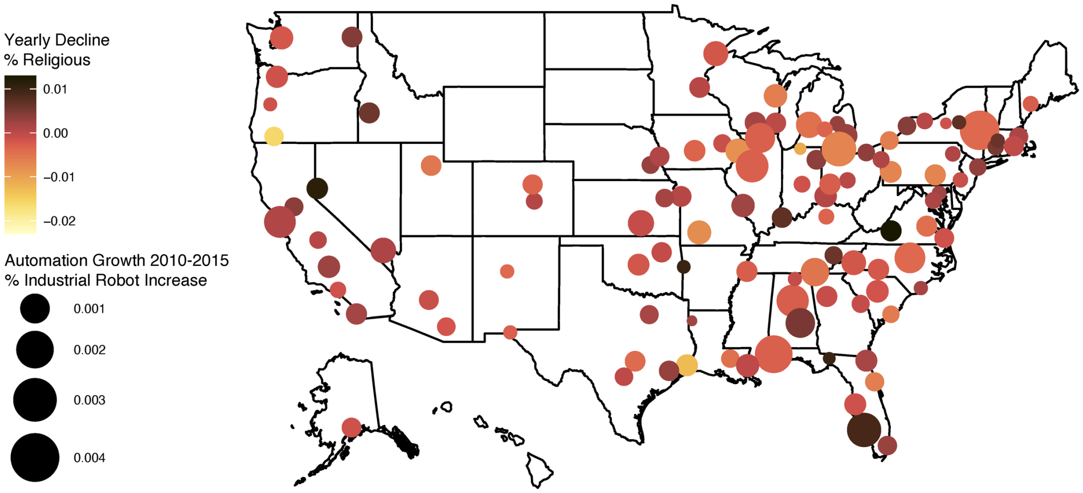

Figure S2. Robotics Growth and Religious Decline across USA Metropolitan Areas. Node color indicates yearly decline in the percent of religious people. Node size indicates robotics growth.

AI Interest and Religiosity Interest in the United States Over Time

Study 2 tested how robotics growth related to religiosity across various American regions. In this study, as in Study 1, robotics growth was associated with religious decline. However, neither Study 1 nor Study 2 featured intensive longitudinal data, which meant that we could not develop pseudocausal models to test whether time-specific increases in robotics growth preceded time-lagged declines in religiosity. We therefore conducted an exploratory analysis in which we used Google Trends to test for the relationship between interest in AI and interest in religion over ten years (2011-2020).

Google Trends is quickly growing as a research tool for quantifying regional and intertemporal variation in preferences and values. One specific advantage of Google Trends is that it is available at the monthly level, whereas many historical surveys (e.g., Gallup World Poll) and corpora (e.g., Google Books) are typically analyzed at the level of the year. Google Trends has also recently been applied to track changes in religious beliefs. Bentzen (34) recently used variation in searches for “prayer” to claim that religious beliefs increased during the COVID-19 pandemic. “Prayer” was a relatively useful keyword in this study because searches for “prayer” are more likely to come from genuine religious belief rather than academic interest in religion (e.g., Atheists may frequently search for “God”) (34).

We built on Bentzen’s (34) study by tracking variation in prayer interest, and also tracking variation in three keywords which connoted interest in artificial intelligence: “AI,” “coding,” and “computer coding.” To reduce researcher degrees of freedom, we pre-registered the study characteristics (e.g., time window and set of terms) before downloading data and running analyses. Our pre-registration is available at https://osf.io/stby4/. A Cronbach’s alpha analysis confirmed that our AI search-terms showed high internal consistency (α = .88), indicating that months with high search volume for “AI” would also have high search volume for “computer coding” and vice versa. We therefore collapsed our AI search-terms so that we had two time-series representing (a) religion interest, and (b) AI interest. Figure S3 displays these time series over our 10-year sample window.

We conducted two time series analyses to determine whether there was a negative lagged relationship between AI interest and religiosity interest. The first analysis was a pre-whitened cross-correlation which visualized the correlation between these two variables at a variety of different lags (e.g., how do changes in AI interest correlate with changes in religiosity interest 5 months later?). “Pre-whitening” a cross-correlation refers to a process where a time series model is fitted to the *x*-variable, extracted, and then

used to residualized the *y*-variable, which is a procedure that is meant to remove possible spurious lagged effects arising from autocorrelation or other interdependence in the time series. The second analysis was a vectoral autoregression which models both autoregressive effects (how do changes in variable *x* at time *t* predict changes in variable *x* at time *t*+1), and cross-lagged effects (how do changes in variable *x* at time *t* predict changes in variable *y* at time *t*+1). For both models, we determined the maximum lag (12 units of time) based on a data-driven function which determines the best maximum lag based on AIC fit. For our VAR model, we differenced the time series before analyzing their bidirectional relationship, so as to remove any possible monotonic trend which could yield a spurious positive correlation between interest in religiosity and interest in AI.

Figure S3 presents the results of the cross-correlation, and Table S13 presents the results of the VAR model. Both models showed bi-directional lagged effects between interest in religiosity and interest in prayer. Religiosity interest could predict future declines in AI interest at an optimal lag of 7 months, and AI interest could predict future declines in religiosity interest at an optimal lag of 10 months. The coefficients of these negative effects are displayed on the y-axis of Figure S3, and in Table S13. Overall, this analysis supports our Study 2 conclusion that rises in AI exposure predict declines in religiosity using pseudocausal methods of time series analysis. This analysis also adds an interesting wrinkle to that finding; religiosity also appears to foreshadow declines in AI interest over time, consistent with a negative reciprocal relationship. However, this is a highly exploratory finding, and should be taken with more caution than the results we present in the main text.

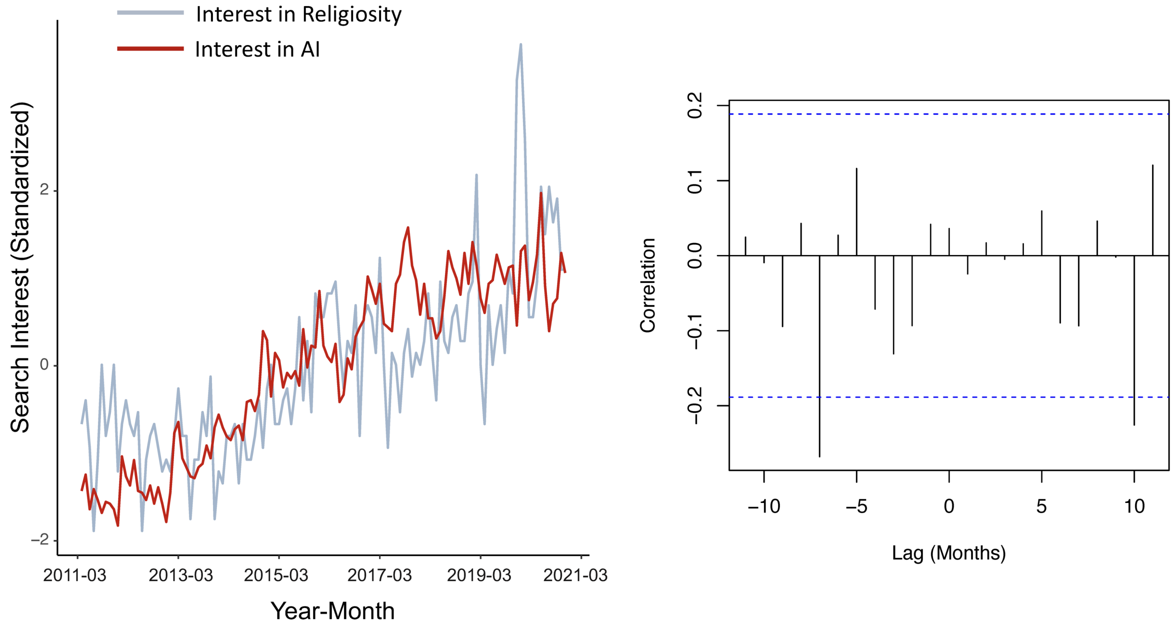

Figure S3. Intertemporal dynamics characterizing interest in AI and interest in religiosity. Left: The raw time-series characterizing interest in prayer (blue) and interest in AI (red). Data are plotted at the monthly level. Right: The results of a pre-whitened cross-correlation between interest in prayer and interest in AI. Each bar represents a correlation at a different lag (bars exceeding the dashed lines are statistically significant), and the lag values *k* are determined on the x-axis. Bars to the left represent religiosity interest predicting AI interest *k*-months in the future; Bars to the left represent AI interest predicting religiosity interest *k*-months in the future.

Table S13.
Lagged Relationship Between Interest in AI and Interest in Religiosity from a VAR Model

Lag	Religiosity Interest Predicting AI Interest	AI Interest Predicting Religiosity Interest
1	.05 (.06)	.01 (.21)

2	-.07 (.06)	-.25 (.23)
3	-.10 (.06)	-.10 (.24)
4	-.08 (.07)	-.37 (.25)
5	.02 (.07)	-.18 (.25)
6	.02 (.07)	-.02 (.25)
7	-.15* (.07)	-.21 (.26)
8	-.09 (.07)	-.06 (.25)
9	-.11 (.07)	-.37 (.25)
10	-.12 (.07)	-.84** (.25)
11	-.05 (.48)	-.08 (.25)
12	-.05 (.38)	-.36 (.24)

Note. Estimates are presented outside parentheses, and standard errors are presented inside parentheses. * $p < .05$; ** $p < .01$; *** $p < .001$.

4. Supplemental Information for Study 3

Descriptive Statistics for Study 3 Variables

Table S14 displays the mean of religious identification and God belief at each wave of Study 3. Table S15 shows the mean of each occupational science exposure variable at each wave of the survey.

Table S14.
Mean of Religiosity Variables at Each Survey Wave

Wave	God Belief	Religious Identification
1	Not Measured	.44
2	.61	.45
3	.52	.40
4	.52	.42
5	.49	.39
6	Not Measured	.40
7	.48	.42
8	.47	.38
9	.45	.36
10	.47	.36
11	.41	.34

Table S15.
Mean of Science Occupational Exposure Variables at Each Survey Wave

Wave	Biology	Chemistry	Mathematics	Medicine	Programming
1	16.94	20.04	51.12	21.07	12.57
2	18.05	20.41	51.25	22.41	12.94
3	17.81	19.20	51.32	21.74	14.05
4	16.39	18.55	50.38	20.62	13.10
5	17.25	18.86	50.54	21.14	13.35
6	17.59	18.99	50.97	21.41	13.89
7	18.02	19.11	50.93	21.89	14.02
8	18.00	19.17	51.14	21.73	14.05
9	18.66	19.33	51.43	22.35	14.55
10	18.19	19.27	51.43	21.60	14.25
11	18.71	19.30	51.46	22.10	14.69

Incorporating Third-Order Lagged Terms in Study 3

In the main text, Table 3 (Model 3) presents a lagged analysis of God belief and occupational AI exposure. We include the first-order and second-order lags, but do not include any higher-order lags

because it reduced our sample to only a fraction (n = 8,262) of the total population. Nevertheless, if we included third-order lags, the third-order lagged effect reached statistical significance, b = -.24, SE = .09, OR = .79, t = -2.70, p = .007, 95% CIs [.67, .94], but with a smaller effect size than the first- and second-order lagged terms (bs = -.24 vs. -.28 and -.71, respectively).

Exact p-Values in Table 3

Table S16.
AI Exposure and God Belief in a Community Sample with Exact p-Values

	Belief in God		
	Estimate (SE)		
	(1)	(2)	(3)
Constant	-2.18*** (p < .001)	-2.22*** (p < .001)	-1.86*** (p < .001)
Timepoint	-0.22*** (p < .001)	-0.22*** (p < .001)	-0.26*** (p < .001)
Income	-0.10*** (p < .001)	-0.17*** (p < .001)	-0.15* (p = .02)
Gender	-2.21*** (p < .001)	-2.10*** (p < .001)	-2.62*** (p < .001)
Age	1.19*** (p < .001)	1.18*** (p < .001)	1.50*** (p < .001)
Conservatism	0.97*** (p < .001)	0.97*** (p < .001)	0.77*** (p < .001)
AI Exposure	-0.53*** (p < .001)	-0.52*** (p < .001)	-0.11 (p = .16)
Biology Exposure		-0.22*** (p < .001)	-0.37** (p = .005)
Chemistry Exposure		-0.05 (p = .34)	-0.03 (p = .74)
Mathematics Exposure		0.05 (p = .08)	0.02 (p = .71)
Medicine/Dentistry Exposure		0.51*** (p < .001)	0.76*** (p < .001)
AI Exposure (lag 1)			-0.26*** (p < .001)
AI Exposure (lag 2)			-0.63*** (p < .001)
Observations	106,956	106,392	30,305
Log Likelihood	-48,817.33	-48,509.15	-12,541.42
Akaike Inf. Crit.	97,654.65	97,046.30	25,114.84
Bayesian Inf. Crit.	97,750.46	97,180.35	25,247.95

Replicating Study 3 Results with Religious Identification Instead of God Belief

Table S17.
AI Exposure and Religious Identification in a Community Sample

	Religious Identification

	Estimate (95% CIs)		
	(1)	(2)	(3)
Constant	-8.87^{***} (-9.09, -8.64)	-8.86^{***} (-9.09, -8.64)	-8.30^{***} (-8.74, -7.87)
Timepoint	-0.10^{***} (-0.11, -0.08)	-0.10^{***} (-0.11, -0.08)	-0.15^{***} (-0.19, -0.12)
Income	0.07^{*} (0.004, 0.13)	0.04 (-0.03, 0.11)	0.08 (-0.06, 0.21)
Gender	-0.42^{***} (-0.57, -0.27)	-0.38^{***} (-0.53, -0.23)	-0.48^{**} (-0.79, -0.17)
Age	0.36^{***} (0.28, 0.45)	0.37^{***} (0.28, 0.45)	0.38^{***} (0.20, 0.56)
Conservatism	0.36^{***} (0.32, 0.40)	0.36^{***} (0.32, 0.40)	0.33^{***} (0.26, 0.40)
AI Exposure	-0.63^{***} (-0.78, -0.48)	-0.60^{***} (-0.74, -0.45)	0.04 (-0.13, 0.21)
Biology Exposure		-0.09 (-0.23, 0.05)	0.07 (-0.18, 0.33)
Chemistry Exposure		-0.004 (-0.11, 0.11)	-0.06 (-0.27, 0.14)
Mathematics Exposure		0.002 (-0.06, 0.07)	0.03 (-0.09, 0.16)
Medicine/Dentistry Exposure		0.18^{***} (0.08, 0.28)	0.02 (-0.16, 0.20)
AI Exposure (lag 1)			-0.05 (-0.21, 0.11)
AI Exposure (lag 2)			-0.47^{**} (-0.77, -0.17)
Observations	119,858	119,219	33,992
Log Likelihood	-48,699.85	-48,479.85	-12,899.54
Akaike Inf. Crit.	97,419.71	96,987.70	25,831.08
Bayesian Inf. Crit.	97,516.65	97,123.34	25,966.03

Note. Estimates are presented outside parentheses, and standard errors are presented inside parentheses. All occupational exposure variables have been standardized via z-scoring for presentation. * $p < .05$; ** $p < .01$; *** $p < .001$.

Additional Robustness Tests for Study 3

We conducted several additional analyses to ensure the robustness of Study 3's findings. These included replicating our central models controlling for general education rather than exposure to specific scientific fields and replicating the relationship between AI exposure and God belief in participants who took part in different subsets of NZAVS waves to control for attrition. We describe each of these approaches below.

Controlling for General Education. Our central models control for occupational exposure to biology, chemistry, mathematics, and medicine/dentistry to ensure that knowledge about science did not confound the relationship between AI exposure and God belief. In supplemental models, we replicated this key relationship controlling for education level as a more general proxy for scientific knowledge. Each participant provided their level of education according to the "New Zealand Qualifications Framework" (NZQF), which provides ten levels ranging from certificates to doctoral degrees communicating different levels of educational qualification. For example, a level 1 certificate communicates "Basic general and/or foundational knowledge" and is earned during secondary education, level 7 indicates "Specialized technical or theoretical knowledge with depth in a field of work or study" and connotes a bachelor's degree, and level 10 indicates "Knowledge at the most advanced frontier of a field of study or professional practice" and connotes a doctoral degree. Simultaneously controlling for education and scientific exposure introduced multicollinearity into our models, so instead we present Table S18 below which controls for general education—scored from 1-10 based on the NZQF codes—rather than exposure to specific scientific fields.

Table S18.
Belief in God Models with General Education Control

	Belief in God

	Estimate (SE)	
	(1)	(2)
Constant	-1.90*** (0.15)	-1.30*** (0.37)
Timepoint	-0.20*** (0.01)	-0.23*** (0.02)
Income	-0.06* (0.03)	-0.05 (0.07)
Gender	-2.24*** (0.08)	-2.76*** (0.19)
Age	1.19*** (0.05)	1.49*** (0.11)
Conservatism	1.01*** (0.02)	0.77*** (0.04)
Education	-0.28*** (0.05)	-0.42*** (0.11)
Occupational Importance of Programming	-0.56*** (0.05)	-0.17* (0.07)
Occupational Importance of Programming (lag 1)		-0.27*** (0.07)
Occupational Importance of Programming (lag 2)		-0.67*** (0.12)
Observations	100,914	28,905
Log Likelihood	-46,499.06	-12,101.85
Akaike Inf. Crit.	93,020.11	24,229.70
Bayesian Inf. Crit.	93,124.86	24,337.23

Note. Estimates are presented outside parentheses, and standard errors are presented inside parentheses. All occupational exposure variables have been standardized via z-scoring for presentation. * $p < .05$; ** $p < .01$; *** $p < .001$.

Exploring Possible Attrition Effects. The models depicted in our main text included all participants, regardless of how many time-points of the NZAVS they completed. To ensure that our effects were not driven by attrition effects, we re-estimated the relationship between occupational AI exposure and God belief with all covariates (see Table 3, Model 2) for participants who reported complete religion and occupation data for (a) at least two time-points, (b) at least three time-points, (c) at least four time-points, (d) at least five time-points, and (e) at least six time-points. Figure S4 shows the effect size of the key relationship between AI exposure and God belief in each of these models, and the error bars represent the standard error in the model. This Figure shows that the effect is statistically significant and similar in magnitude regardless of whether we rule out participants who participated in a smaller fraction of survey waves. We therefore consider it unlikely that attrition drove our findings.

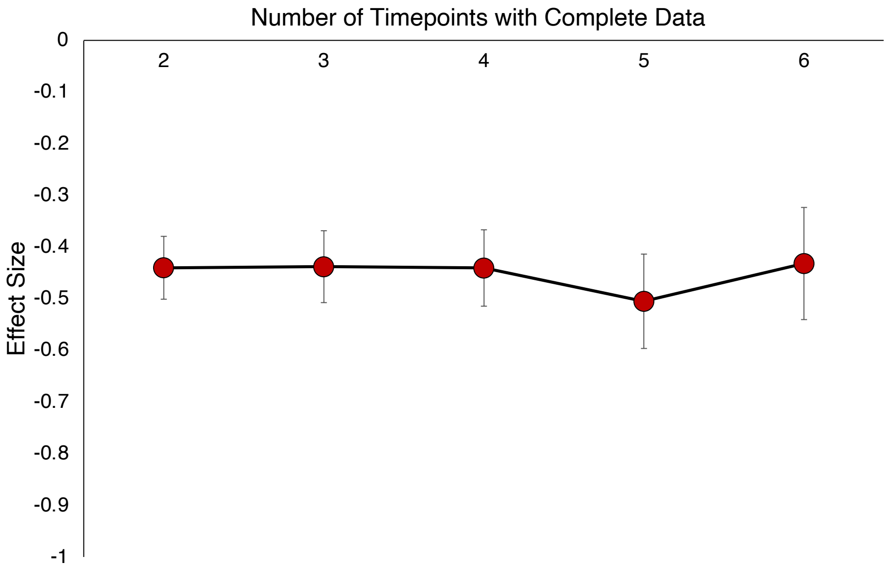

Figure S4. The relationship between AI exposure and God belief across participants who completed varying number of time-points. Nodes represent the effect size. Error bars represent standard error. These estimates control for age, gender, income, timepoint, political conservatism, and exposure to other scientific disciplines.

Ruling out Alternative Modeling Strategies in the Study 3 Dataset

We considered a multi-level modeling approach in which we interacted each person's mean AI exposure with survey timepoint. This approach would be similar to Studies 1-2, which tested whether countries and states with high levels of robotics experienced greater religious decline throughout the 21st century. However, the approach is less appropriate for Study 3 because countries and states almost never experience declining automation, whereas individuals frequently shift between jobs which have different levels of exposure to AI. This means that someone's mean level of AI exposure across the entire NZAVS survey is not a very informative statistic. We therefore considered it more appropriate to track how a person's occupational AI exposure at a given time-point *t* correlated with their religiosity at that time-point, and also their religiosity at future time-points in the survey using lagged terms. We also considered (and pre-registered as an alternative analysis) a cross-lagged panel model with random intercepts. However, this model did not converge, so we elected to use multi-level models (which we also pre-registered) to test our hypotheses

5. Supplemental Information for Study 4

Study 4 Workplace Behavior Measures

Our main text focuses on the measures in timepoint 1 (T1) and timepoint 2 (T2) which test our main hypothesis. But we also investigated the downstream consequences, measured in timepoint 3 (T3), of a negative association between automation and religiosity in our Study 4 dataset. Past research has linked religiosity to a host of positive outcomes. Studies on religion and social behavior have found that religious people often behave more prosocially (35,36) and honestly (37,38), and that they are trusted more than non-religious people (39,40). Other studies have described more cognitive correlates of religiosity, such as higher levels of self-control (41,42) and conscientiousness (43). If automation leads to religious decline, could it also encourage declines in these positive attributes?

We measured workplace behaviors that resembled outcomes previously linked to religiosity in previous research on religion and prosociality (35,39,44) (e.g., unethical behavior, trust, incivility, organizational citizenship behavior, which are face-valid indicators of prosociality) and on religion and cognitive control (41,42) (e.g., goal progress, task proficiency, which have been previously linked to self-control (45)). The fact that these measures were supervisor-reported helped mitigate self-report biases such as social desirability concerns (46). Finally, we measured age, gender, socioeconomic status, education, and tenure with the company as pre-registered covariates.

These workplace behavior measures are listed below, and Table S19 displays the descriptive statistics of all measures.

Goal Progress (T3). We used the Goal Progress scale developed by Wanberg, Zhu, & Van Hooft (47). Each participant was rated by their supervisor on a 1 (Strongly agree) - 5 (Strongly disagree) scale across 6 items, including "___ was productive at work" and "___made good progress at work". Each item started with "Over the last week" to make the scale sensitive to the context of the data collection.

Task Proficiency (T3). We used the Task Proficiency scale developed by Mitchell and colleagues (48). Each participant was rated by their supervisor on a 1 (Strongly agree) - 5 (Strongly disagree) scale across three items, including "___ carried out the core parts of his/her job well" and "___ completed his/her core tasks well using the standard procedures." Each item started with "Over the last week" to make the scale sensitive to the context of the data collection.

Organizational Citizenship Behavior (T3). We used the Organizational Citizenship Behavior scale developed by Lee and Allen (49). Each participant was rated by their supervisor on a 1 (Very low) to 7 (Very high) scale across three items, including “Overall level of effort of ___” and “Overall willingness to do what it takes to successfully complete assigned tasks of ___.” Each item started with “Over the last week” to make the scale sensitive to the context of the data collection.

Counterproductive Workplace Behavior (T3). We used the Counterproductive Workplace Behavior scale developed by Bennett & Robinson (50). Each participant was rated by their supervisor on a 1 (Strongly disagree) to 5 (Strongly agree) scale across seven items from Bennett & Robinson’s (2000) measure of counterproductive workplace behavior. Items highlighted various behaviors such as cursing, pranking, or publicly embarrassing colleagues for ethical failings at work.

Instigated Incivility (T3). We used the Instigated Incivility scale used in Koopman and colleagues (51). Each participant was rated by their supervisor on a 1 (Strongly disagree) to 5 (Strongly agree) scale across three items, including “___ put a co-worker down or acted condescendingly towards them” and “___ paid little attention to a coworker’s statement or showed little interest in their opinion.” Each item started with “Over the last week” to make the scale sensitive to the context of the data collection.

Unethical Behavior (T3). We used the Unethical Behavior scale used in Welsh, Bush, Thiel, and Bonner (52). Each participant was rated by their supervisor on a 1 (Strongly disagree) to 5 (Strongly agree) scale across four items, including “___cuts corners to complete work assignments more quickly” and “___ alters performance numbers to appear more successful.” Each item started with “Over the last week” to make the scale sensitive to the context of the data collection.

Perceived Trust (T3). We used the Perceived Trust scale developed by Robinson (53). Each participant was rated by their supervisor on a 1 (Strongly disagree) to 5 (Strongly agree) scale across six items, including “I believed that ___ has high integrity” and “___ was open and upfront with me.”

Table S19.
Descriptive Statistics for Variables in Study 4

Variable	Mean	Standard Deviation
Intrinsic Religiosity (T1)	3.13	.65
Religious Fundamentalism (T1)	3.35	.57
Occupational AI Exposure (T1)	3.05	.84
Religiosity (T2)	3.55	.81
Goal Progress (T3)	3.74	.69
Task Proficiency (T3)	3.67	.84
Organizational Citizenship Behavior (T3)	3.84	.59
Counterproductive Workplace Behavior (T3)	3.15	.89
Instigated Incivility (T3)	2.85	1.00
Unethical Behavior (T3)	2.48	.91
Perceived Trust (T3)	3.13	.50

Study 4 Full Multiple Regression Models

Our main text reports the results of regression models in which T1 AI exposure was negatively associated with T2 religiosity, even controlling for T1 religious fundamentalism and intrinsic religiosity. In Table S20, we summarize the full statistics associated with this relationship.

These models also found an interaction between T1 AI exposure and T1 intrinsic religiosity, such that the negative link between T1 AI exposure and T2 religiosity was stronger for participants who began the study lower in intrinsic religiosity compared to those who began the study higher in intrinsic religiosity. Table S19 displays the coefficients from these regression models. These models also control for age, gender, education, SES, and organizational tenure, which were our pre-registered covariates.

Table S20.

Full Coefficients for Study 4 Multiple Regression Models						
	df	Adj. R^2	*b*(SE)	*β*	*t*	*p*
**Model 1: Main Effects**	229	.04				
AI Exposure			-.18 (.08)	-.19	-2.17	.03
Intrinsic Religiosity			.13 (.11)	.10	1.18	.24
Religious Fundamentalism			.08 (.09)	.06	.88	.38
Age			-.003 (.007)	-.03	-.46	.65
Gender			.04 (.11)	.03	.39	.70
Education			.06 (.06)	.07	1.04	.30
SES			< .001 (.02)	.002	.04	.97
Organizational Tenure			.01 (.03)	.03	.38	.70
**Model 2: One Interaction**	229	.06				
AI Exposure			-.19 (.08)	-.20	-2.30	.02
Intrinsic Religiosity			.14 (.11)	.11	1.31	.19
Religious Fundamentalism			.08 (.09)	.06	.91	.37
Age			-.004 (.006)	-.04	-.60	.55
Gender			.04 (.11)	.02	.35	.73
Education			.05 (.06)	.05	.80	.42
SES			-.001 (.02)	-.004	-.06	.95
Organizational Tenure			.02 (.03)	.05	.63	.53
AI Exposure * Intrinsic Religiosity			.18 (.08)	.14	2.19	.03
**Model 3: Both Interactions**	227	.06				
AI Exposure			-.17 (.08)	-.18	-2.06	.04
Intrinsic Religiosity			.15 (.10)	.12	1.44	.15
Religious Fundamentalism			.09 (.09)	.06	.95	.35
Age			-.004 (.007)	-.05	-.64	.53
Gender			.03 (.11)	.02	.26	.80
Education			.05 (.06)	.05	.82	.41
SES			-.001 (.02)	-.002	-.03	.97
Organizational Tenure			.02 (.03)	.05	.69	.49
AI Exposure * Intrinsic Religiosity			.17 (.08)	14	2.10	.04
AI Exposure * Religious Fundamentalism			.14 (.11)	.08	1.28	.20

Study 4 Supplemental Measures

With these data, we were able to test whether AI-linked declines in religiosity predict subsequent variation in workplace behaviors. We did this using a structural equation model. We began by modeling the pre-registered mediation dynamic (AI exposure → religiosity → workplace behaviors) and then adding additional paths using a data-driven approach with modification indices. We also chose to incorporate intrinsic religiosity as a moderator of the AI exposure → religiosity path given the significant interaction we observed in our initial models.

Table S21 summarizes the model-building process. Model 1 was our original model, which only modeled the relationship between AI exposure and religiosity, the interaction between AI exposure and intrinsic religiosity on religiosity, and the relationships between religiosity and workplace behaviors. This model showed poor initial fit, with a significant chi-squared statistic, CFI and TLI values below .95, and an RMSEA value above .05. We used modification indices in a data-driven approach which identified the paths which would improve model fit if they were included in the equation. These included (a) adding a relationship between intrinsic religiosity and organizational citizenship behavior (MI = 15.75), (b) adding a relationship between AI exposure and unethical behavior (MI = 16.19), (c) adding a relationship between AI exposure and trust (MI = 7.61), and (d) adding a relationship between AI exposure and task proficiency (MI = 7.70). Every MI-derived pathway resulted in significant improvement in model fit, as estimated

through likelihood ratio tests which produced significant improvements in chi-squared fit. The final model (Model 5) showed no modification indices above 5.00 and showed good model fit across all fit statistics. Table S22 summarizes the variances and covariances in this final model.

Table S21.
Model-Building in Study 4

Model	DF	Chi Squared	Δ Chi Squared	CFI	TLI	RMSEA	Largest MI
1	56	χ^2= 111.52, p < .001		.81	.69	.07	Intrinsic Religiosity → Org. Citizen. Behavior
2	55	χ^2= 90.07, p = .002	χ^2= 21.45, p < .001	.88	.80	.05	AI Exposure → Unethical Behavior
3	54	χ^2= 71.61, p = .06	χ^2= 18.47, p < .001	.94	.90	.04	AI Exposure → Trust
4	53	χ^2= 63.39, p = .16	χ^2= 8.22, p = .004	.97	.94	.03	AI Exposure → Task Proficiency
5	52	χ^2= 55.38, p = .35	χ^2= 8.00, p = .005	.99	.98	.02	None above 5.00

Table S22.
Variances Covariances in Final Study 4 Model

Statistic	Variable	Estimate (SE)	***p*-value**
Variances	Religiosity	.60 (.06)	< .001
	Organizational Citizenship Behavior	.30 (.04)	< .001
	Goal Progress	.45 (.04)	< .001
	Counterproductive Workplace Behaviors	.71 (.06)	< .001
	Instigated Incivility	.95 (.07)	< .001
	Unethical Behavior	.59 (.06)	< .001
	Task Proficiency	.65 (.06)	< .001
	Trust	.24 (.02)	< .001
Covariances	Organizational Citizenship Behavior ~~		
	Goal Progress	.03 (.03)	.22
	Counterproductive Workplace Behaviors	-.06 (.03)	.05
	Instigated Incivility	-.13 (.03)	.001
	Unethical Behavior	-.07 (.03)	.02
	Task Proficiency	.04 (.03)	.21
	Trust	.009 (.02)	.60
	Goal Progress ~~		
	Counterproductive Workplace Behaviors	-.07 (.04)	.07
	Instigated Incivility	-.08 (.04)	.05
	Unethical Behavior	-.07 (.03)	.05
	Task Proficiency	.02 (.04)	.52
	Trust	-.002 (.02)	.91
	Counterproductive Workplace Behaviors ~~		
	Instigated Incivility	.12 (.06)	.04
	Unethical Behavior	.25 (.05)	< .001
	Task Proficiency	-.06 (.05)	.20
	Trust	.09 (.03)	.003
	Instigated Incivility ~~		
	Unethical Behavior	.19 (.05)	< .001
	Task Proficiency	-.06 (.05)	.27
	Trust	.03 (.03)	.30
	Unethical Behavior ~~		
	Task Proficiency	-.18 (.04)	< .001
	Trust	.04 (.03)	.09
	Task Proficiency ~~		
	Trust	.002 (.03)	.94

This SEM (see Figure S5) reproduced the negative lagged association between T1 AI exposure and T2 religiosity, as well as the moderation by T1 intrinsic religiosity. It also reproduced many of the associations documented in previous research involving prosociality and cognitive control. Religiosity was positively associated with organizational citizenship behavior, goal progress, and task proficiency, and was negatively associated with counterproductive workplace behavior, unethical behavior, and incivility. Past studies have shown that religious people can show pronounced social desirability biases in self-report surveys (54), but we measured T3 workplace behavior using supervisor-report, increasing the dependability of these associations.

Our model also revealed two unexpected findings. First, we found that T2 religiosity was not associated with T3 perceived trust. Previous research on religion and trust has sampled people living in religious communities (39,55) whereas our sample of employees in a manufacturing plant may not have relied on religion to the same extent to gauge trust. Second, AI exposure was linked to T3 supervisor-reports of higher perceived trust, task proficiency, and lower unethical behavior. This suggests that, at least in the workplace, adopting AI technology can carry benefits, leading employees to be perceived as more proficient, ethical, and trustworthy. However, AI exposure showed no significant association with supervisor-reports of workplace behaviors, such as organizational citizenship, goal progress, counterproductive workplace behavior, or incivility, suggesting that it had a narrower range of benefits compared to religious belief. Table S22 summarizes these statistics in full.

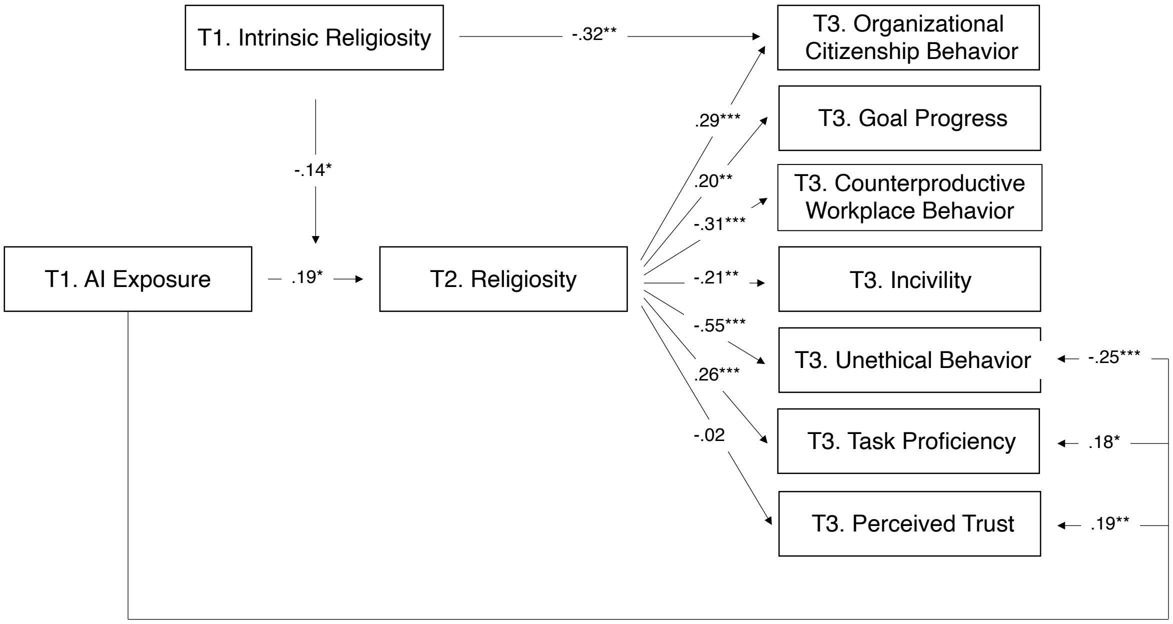

Figure S5. A Structural Equation Model Displaying Study 4 Results. AI exposure and intrinsic religiosity have been centered. Coefficients have been standardized and can be interpreted as effect sizes. Variances and covariances are not shown here for display purposes, but they are listed in Table S22. This model includes covariates (age, gender, SES, education, tenure in organization) which are omitted for display purposes. * $p < .05$; ** $p < .01$; *** $p < .001$.

6. Supplemental Information for Study 5

Stimuli in Study 5

In our main text Methods, we summarize the experimental design of Study 5, which involved reading about three innovations in language, medicine, and agriculture. Table S23 summarizes the paragraphs

that participants read about each advance, and provides a link to the source of each paragraph. Some paragraphs have been slightly adapted for spelling, grammar, and length.

Table S23.
Stimuli in Study 5

Condition, Domain	Text, Source
AI, Language	ChatGPT is a natural language processing tool that allows users to have human-like conversations with an AI chatbot. Users can ask all kinds of questions to ChatGPT to get straightforward and uncluttered responses in return. For example, you can use the tool as an encyclopedia and ask questions. For instance, “define Newton’s laws of motion” or “write a poem,” which it will do instantly. Additionally, you may ask ChatGPT to design a computer program which performs simple or complex tasks such as solving anagrams or detecting animals using data about their average height and weight. ChatGPT is so good at emulating human language that many people cannot distinguish between ChatGPT and written responses by real people.<br><br>Source:<br>https://emeritus.org/blog/ai-ml-what-is-chatgpt/
AI, Medicine	Sharing medical data between laboratories and medical experts is important for medical research. However, data sharing is often sufficiently complex and sometimes even impossible due to the strict data regulatory legislation in Europe. Researchers addressed the problem and developed an artificial neural network that creates synthetic x-ray images that can fool even medical experts.<br><br>Source: https://www.sciencedaily.com/releases/2022/11/221117102821.html
AI, Agriculture	Internet of Things (IoT) is a new method of giving physical objects "minds," with sensors, memory, processing ability, and communication ability. These devices can communicate with each other, adapt their behavior, and predict the future without an internet connection. IoT is revolutionizing agriculture because it allows surveillance cameras, tractors, sprinklers, and other agricultural tools to exchange data on temperature, humidity, waste, wind speed, and pest infestation in order to control plant treatment (e.g., including pesticide control and water volume) without farmers actively making decisions.<br><br>Source:<br>https://www.forbes.com/sites/louiscolumbus/2021/02/17/10-ways-ai-has-the-potential-to-improve-agriculture-in-2021/?sh=3f5727f17f3b
Science, Language	Over 70 million deaf people use sign languages as their preferred communication form. Although they access similar brain structures as spoken languages, it hasn't been identified the brain regions that process both forms of language equally. Scientists have now discovered that Broca's area in the left hemisphere, central for spoken languages, is also crucial for sign languages. This is where the grammar and meaning are processed, regardless of whether it is spoken or signed language.<br><br>Source:<br>https://www.sciencedaily.com/releases/2022/12/221220112426.htm
Science, Medicine	Fewer cases of melanoma were observed among regular users of vitamin D supplements than among non-users, a new study finds. People taking vitamin D supplements regularly also had a considerably lower risk of skin cancer, according to estimates by experienced dermatologists. The study included nearly 500 people with an increased risk of skin cancer.

	Source: https://www.sciencedaily.com/releases/2023/01/230109112555.htm
Science, Agriculture	A research group is studying how plants 'breathe'. They have gained new insights into how grasses develop efficient 'breathing pores' on their leaves. If important landmark components in this development process are missing, the gas exchange between plant and atmosphere is impaired. These findings could nonetheless be relevant to improving agricultural crops.<br><br>Source: https://www.sciencedaily.com/releases/2022/12/221223103421.htm

Robustness Analyses for Study 5

Here we report two robustness analyses for Study 5. The first series of analyses reports the main effects of Study 5 without controlling for perceived impressiveness and technological sophistication. Our second series of analyses reports the main effects of Study 5 without including participants from our pilot data. Results were substantively identical to our main text analyses in both cases.

Without controlling for impressiveness and technological sophistication, participants viewed AI advances as less associated with laws of nature than scientific innovations, $b = -1.44$, $SE = 0.07$, $t = -20.59$, $p < 0.001$, *95% CIs* [-1.58, -1.30]. We also found that participants reported less religious conviction in the AI condition vs. the science condition, $b = -0.43$, $SE = 0.09$, $t = -4.90$, $p < 0.001$, *95% CIs* [-0.61, -0.26].

When excluding subjects from our pilot study, participants viewed AI advances as less associated with laws of nature than scientific innovations, $b = -1.63$, $SE = 0.09$, $t = -17.95$, $p < 0.001$, *95% CIs* [-1.81, -1.45]. We also found that participants reported less religious conviction in the AI condition vs. the science condition, $b = -0.73$, $SE = 0.12$, $t = -6.24$, $p < 0.001$, *95% CIs* [-0.95, -0.50].

7. Study S1

We ran this study during a 7-hour MBA seminar in Singapore, in which senior executives from Taiwan learned about workplace applications of AI. The module introduced easy-to-use computing techniques in R and Python for implementing AI machine learning solutions. We randomly assigned participants to either the control or experimental condition. Participants in the control condition ($n = 35$) received their questions at the beginning of the seminar (before AI exposure); participants in the experimental condition ($n = 43$) received their questions at the end of the seminar (after AI exposure). Among participants who reported their gender, there were 27 men and 30 women, and a median age of 40 – 50.

The first questions in the survey were a manipulation check measuring whether attending the seminar actually increased confidence in automation (we reasoned that even anticipating the seminar could increase these perceptions). Participants separately rated the promise of automation (AI and robotics), medicine, biology, chemistry, and mathematics with a 1 ("No Promise") – 7 ("Very Promising") scale. Participants then rated three "playing God" items using a 1 ("Strongly Disagree") – 7 ("Strongly Agree") scale. These items were: (a) Artificial intelligence and robotics allow humans to "break" the laws of nature, (b) Artificial intelligence and robotics allow humans to do things that we have never been able to do before, (c) Artificial intelligence and robotics give humans "superhuman" abilities. Finally, participants rated three items measuring religiosity: (a) Belief in God has an important role in the workplace, (b) Prayer has an important role in the workplace, and (c) Religious service attendance has an important role in the workplace. Participants also reported their religious identity and rated the importance of religion in their life using a 1 (Not at all Important) – 7 (Very Important) scale during the demographics section of the survey.

The manipulation succeeded such that participants in the AI condition rated AI as more promising for the future than participants in the control condition, $b = 0.38$, $SE = 0.17$, $t = 2.19$, $p = 0.03$, but did not rate

any other scientific discipline as more promising for the future (*p*s > 0.05). The manipulation also increased participants' confidence that they could "play God" with AI technology. Participants in the experimental condition agreed significantly more with the items "Artificial intelligence and robotics allow humans to "break" the laws of nature," $b = 1.18$, $SE = 0.44$, $t = 2.67$, $p = 0.009$, "Artificial intelligence and robotics allow humans to do things that we have never been able to do before," $b = 0.93$, $SE = 0.32$, $t = 2.93$, $p = 0.005$, and marginally more with the item "Artificial intelligence and robotics give humans 'superhuman' abilities," $b = 0.64$, $SE = 0.36$, $t = 1.79$, $p = 0.08$. See Figure S6 for an illustration of these effects.

There was no significant main effect of experimental condition on any of the three religion items (*p*s > 0.05). However, condition interacted with religious importance on ratings of prayer, $b = -0.52$, $SE = 0.17$, $t = -3.00$, $p = 0.004$, and service attendance, $b = -0.55$, $SE = 0.17$, $t = -3.22$, $p = 0.002$. For participants who rated religion as not at all important in their life, the AI seminar did not have a significant effect on these items (*p*s > 0.05). But for participants who rated religion as very important in their life, the AI seminar decreased perceived importance of prayer at work, $b = -0.82$, $SE = 0.32$, $t = -2.57$, $p = 0.01$, and service attendance at work, $b = -1.00$, $SE = 0.31$, $t = -3.22$, $p = 0.002$. Figure S6, breaks down the effect of condition at each level of religious importance to display this moderation.

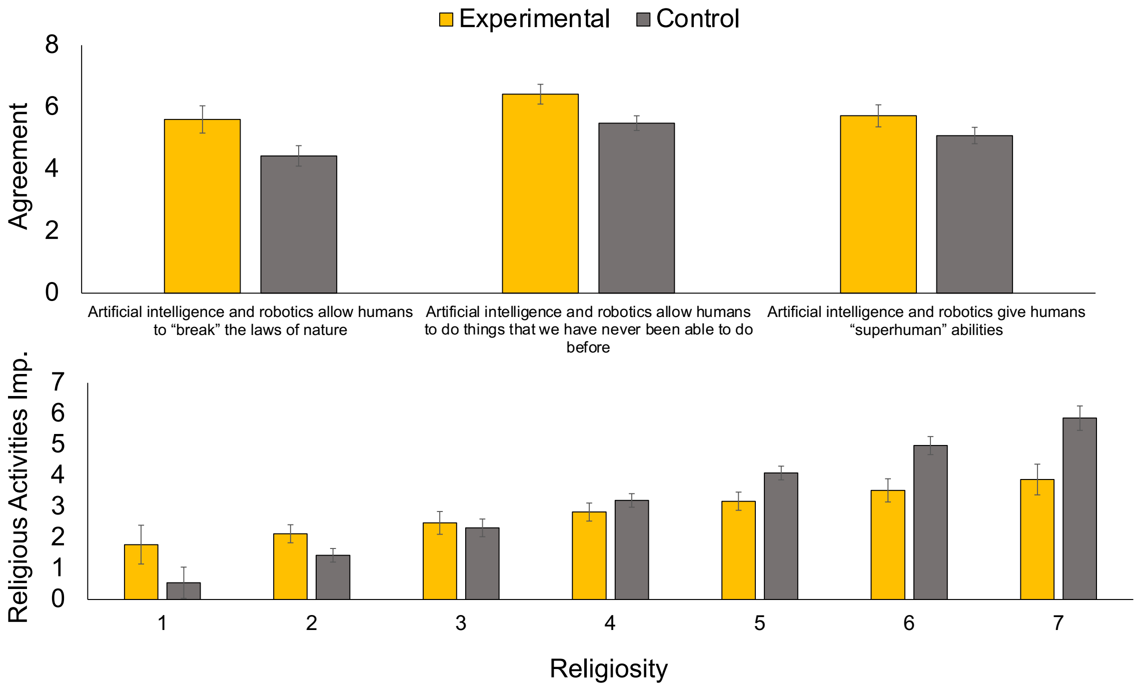

Figure S6. Top) Estimates of playing God items in the control and experimental conditions from a general linear model. Bottom) Estimates of religious activities (prayer and service attendance) importance in the workplace from a general linear model where condition is moderated by religiosity. We present prayer and services together because their effects were nearly identical.

In sum, this field experiment showed that exposure to AI through an intensive one-day seminar increased senior business executives' belief that artificial intelligence allows humans to "play god," break the laws of nature, and do things that humans have never done before. Among highly religious individuals, exposure to AI also decreased the importance of prayer and service attendance for workplace behavior, although it did not change perceptions of God's importance. Nevertheless, the items measuring prayer and service attendance are interesting in their own right, given these are two forms of petitionary religious appeals.

Despite this study's small sample, it offers valuable evidence that automation leads people to feel unconstrained by laws of nature, and that exposure to automation may reduce people's perceived importance of religious appeals—at least among the highly religious.

8. Study S2a-b

In two highly related supplemental studies (Studies S2a-b), we examined the cross-sectional relationship between AI favorability and religiosity in two different datasets. We reasoned that non-religious people would be more favorable towards automation than religious people for at least two reasons. First, favorability towards automation may lead to religious decline (producing a negative correlation with religiosity). Second, religious people may perceive automation as more of a threat to their worldview than non-religious people.

We examined this relationship with two datasets. The first "international" dataset (Study S2a) consisted of data collected by the Pew Research Center between 2019-2020 from 32,330 people (16,890 men, 15,440 women; M_{age} = 48.45, SD_{age} = 18.12) across Australia, Brazil, Canada, Czech Republic, France, Germany, India, Italy, Japan, Malaysia, the Netherlands, Poland, Russia, Singapore, South Korea, Spain, Sweden, Taiwan, United Kingdom, and the United States. Table S24 displays the religious demographic of the sample. In this dataset, not all participants answered all questions (some participants indicated responses of "don't know" or "no opinion" which were scored as missing in our analyses), so degrees of freedom vary across analyses.

Table S24.
Religious Identification of Participants in the Study S2a

Country	Christian	Muslim	Baha'i	Hindu	Buddhist	Sikh	Jewish	None	Other
Australia	726	23	2	30	26	2	3	611	99
Brazil	1257	0	0	0	0	0	1	102	114
Canada	880	34	0	21	13	15	23	438	90
Czech Republic	470	0	0	0	3	0	3	883	24
France	719	76	0	0	13	0	7	580	26
Germany	849	42	0	0	9	0	2	581	16
India	76	275	0	2675	41	54	0	9	20
Italy	1124	16	0	2	0	0	0	289	43
Japan	21	1	0	1	548	0	0	894	5
Malaysia	146	1144	1	75	228	2	0	30	16
Netherlands	636	38	0	11	21	0	2	712	75
Poland	1386	0	0	0	0	0	0	71	11
Russia	1031	148	0	0	3	0	0	340	185
Singapore	347	142	0	142	375	0	1	393	58
South Korea	533	0	0	0	249	0	0	771	9
Spain	827	16	0	0	0	0	1	244	41
Sweden	869	22	0	0	0	0	0	615	59
Taiwan	163	1	0	0	518	0	0	384	484
United Kingdom	731	62	0	12	11	7	3	606	33
United States	923	8	0	8	7	0	50	337	103

Note. Values marked "NA" were not read to participants. "Christian" includes participants who reported being Roman Catholics, Protestants, Orthodox, and "just a Christian." "None" includes participants who reported being Atheists, Agnostics, and "Nothing in Particular." Participants who refused to answer the question or answered "Don't Know" are not included in this table.

The second "USA" dataset (Study S2b) consisted of publicly available data collected by the Pew Research Center in 2017 which comprised of 4,135 people from the United States (2,046 men, 2,089 women; 463 participants aged 18-29, 1177 aged 30-49, 1331 aged 50-64, 1160 aged 65+; 2,648 Christians, 92 Mormons, 143 Jews, 29 Muslims, 33 Buddhists, 27 Hindus, 1,069 Non-Religious, 1 refused to answer). This survey also included information about political party lean (772 people leaned

Republican, 714 people leaned Democrat) and college attainment (2,183 participants had earned their college degree).

Both datasets contained measures of people's AI favorability. In the international dataset, participants rated whether (a) robots and (b) AI was a good or bad thing for society using a binary scale. We chose to average these responses into a composite variable because the responses to the two items also correlated strongly, $r(25{,}102) = .45$, $p < .001$, and using a composite of the two items gave us more statistical power than if we had used either item in isolation since participants did not always respond to all items. In the USA dataset, participants rated their enthusiasm about "the possibility that computers and robots could do most of the work currently done by humans," using a scale of 1-4 anchored at 1 ("Very enthusiastic") and 4 ("Not at all enthusiastic"). We reverse-scored this item so that higher values represented greater enthusiasm.

Both datasets also contained measures of religiosity. In the international dataset, participants responded to the item "how important is religion in your life," which was rated on a 1-4 scale anchored at 1 ("Very Important") to 4 ("Not at all important"). We reverse-scored the scale so that higher values represented greater importance. In the USA dataset, participants rated their frequency of service attendance using a 1-6 scale ranging from "never" to "more than once per week." These different measures allowed us to test how attitudes towards AI were linked to religious behaviors as well as self-reported religious importance.

Our international dataset also allowed us to measure people's favorability towards science more generally, and other technological innovations using the same binary scale that they used to rate robots and AI. Specifically, participants rated their favorability towards science with the item "Overall, would you say developments in science have had a mostly ____ effect on society?" with response options ranging from (1) mostly positive effect, (2) mostly negative effect, and (3) equal positive and negative effects. We recoded these options so that values were ordered in terms of positivity, which 3 representing "mostly positive effect" and 1 representing "mostly negative effect."

How did people's attitudes about AI relate to their religiosity? A zero-order correlation found that AI favorability was negatively linked with religiosity in the international dataset, $r(30{,}202) = -.08$, $p < .001$. This negative correlation remained after controlling for sex, age, favorability towards science, and favorability towards space travel in a regression model where intercepts randomly varied across nations (Table S25, Model 1). Favorability towards science was negatively associated with religiosity, but its association was far weaker than the negative association between AI favorability and religiosity. Favorability towards space travel was *positively* associated with religiosity in the model. Similarly, in the USA dataset, AI favorability was negatively associated with religiosity, $r(4{,}110) = -.09$, $p < .001$, and this negative association persisted controlling for age, gender, education, and political orientation (Table S25, Model 2). Participants from around the world and across the United States with more favorable attitudes towards AI had lower levels of religiosity, and this association could not be reduced to education, age, gender, political orientation, or favorability towards science and technology.

Table S25.
AI Favorability and Religiosity Across Individuals Around the World and in the USA

Model | Predictor	*b (SE)*	*β*	*t*	*p*	95% LLCI	95% ULCI
**International Dataset (Study S2a)**						
AI Favorability	-.16 (.016)	-.06	-10.18	< .001	-.20	-.13
Male	-.17 (.01)	-.07	-13.49	< .001	-.20	-.15
Age	.008 (.0004)	.12	20.96	< .001	.007	.008
Science Favorability	-.02 (.01)	-.01	-2.18	.03	-.04	-.002
Space Favorability	.04 (.02)	.01	2.16	.03	.004	.08
**USA Dataset (Study S2b)**						
AI Favorability	-.21 (.06)	-.10	-3.74	< .001	-.32	-.10
Male	.12 (.04)	.07	2.94	.003	.04	.20
Age	-.27 (.09)	-.08	-3.06	.002	-.44	-.10
College Education	.15 (.09)	.05	1.77	.08	-.02	.32
Republican	.80 (.09)	.24	9.28	< .001	.63	.97

Note. The Study S2a model comes from a multilevel model with intercepts varying randomly across nations. The Study S2b model comes from a general linear model of American participants. Predictors are indented below model titles.

9. Study S3

In Study S3, we tested whether religious individuals perceived religion and automation as compatible. A well-established finding in research on science and religion is that religious people view science and religion as highly compatible (56,57). This is because people view science and religion as fulfilling different capacities and meeting different needs. Science involves the human exploration and application of laws of nature, whereas religion involves "supernatural" agents and principles that transcend these laws (58)[1]. We predicted that, because many people also believe that automation can operate outside these laws of nature, religious people would view religion as less compatible with automation than science.

We designed a pre-registered within-subjects experiment in which 498 religious individuals rated AI, robotics, and other branches of science (biology, chemistry, mathematics, medicine, the same disciplines that we measured in Study 3) on 12 bipolar and unipolar items: The bipolar items were (1) "The field of ___ is focused on HOW [vs. WHY] to solve problems" (2) "The field of ___ is focused on concrete observable information [vs. abstract ideas and principles]," and (3) "The field of ___ is focused on abstract ideas and principles [vs. intuition]." The unipolar items, anchored at 1 ("Strongly Disagree") and 7 ("Strongly Agree"), were (4) "The field of ___ involves agents with cognitive or physical abilities that surpass human abilities," (5) "People who work in ___ are playing God," (6) "People who work in ___ are doing things that should be left to God," (7) "The field of ___ involves providing social support to people," (8) "The field of ___ involves providing connection to a community," (9) "The field of ___ involves applying laws of nature," (10) "The field of ___ involves discovering laws of nature," (11) "God works through ___ ," (12) "Religion is compatible with ___."

We pre-registered five latent factors underlying these items, and a promax factor analysis suggested that five factors each explained > 10% of variance in the items, with a cumulative variance explained of 69%. As pre-registered, there was a construal level factor (items 1-3), a playing god factor (items 4-6), a communality factor (items 7-8), a laws of nature factor (items 9-10), and a compatibility with religion factor (items 11-12). All item loadings were greater than .30, with no cross-loadings above .30. We averaged the items into these five indices for analyses.

After computing these indices, we fit a multilevel model in which each dimension was regressed on discipline dummy-codes, contrasted against automation (the average score of AI and robotics). We combined robotics and AI into a single index for the sake of parsimony, but it had little impact on our findings: Our results replicated regardless of whether we modeled robotics or AI individually.

Our first model found that people rated automation as less compatible with religion than all other scientific disciplines (see Table S26). We next investigated the possible mechanisms of this effect by examining whether automation was unique from all other disciplines in other respects. Our models showed that automation was not viewed as significantly different from other disciplines in terms of prosociality, or construal level (*p*s > .10). However, automation was seen as less associated with laws of nature compared to any other scientific discipline. Automation was also seen as significantly more associated with playing God than all other disciplines. Statistics from these key models are displayed in Table S26.

Table S26.
Key Properties of Automation vs. Scientific Disciplines in Study S3

[1] This distinction between the "natural" and "supernatural" is often fuzzy in practice, because people view gods and spirits as exercising their supernatural powers through nature (59). However, many cultures appear to hold the belief that God(s) can regularly violate laws of physics, biology, psychology, and chemistry that regulate the natural world (13).

Outcome, Contrast	*b*	*SE*	*t*	*p*	95% CIs
**Compatible With Religion**					
Biology vs. Automation	1.07	0.06	19.29	< 0.001	0.96, 1.18
Chemistry vs. Automation	0.82	0.06	14.73	< 0.001	0.71, 0.92
Mathematics vs. Automation	0.62	0.06	11.27	< 0.001	0.52, 0.73
Medicine vs. Automation	1.38	0.06	24.84	< 0.001	1.27, 1.49
**Associated With Laws of Nature**					
Biology vs. Automation	1.38	0.06	21.94	< 0.001	1.26, 1.51
Chemistry vs. Automation	1.15	0.06	18.19	< 0.001	1.02, 1.27
Mathematics vs. Automation	0.20	0.06	3.22	0.001	0.08, 0.33
Medicine vs. Automation	1.30	0.06	20.69	< 0.001	1.18, 1.43
**Encourages Playing God**					
Biology vs. Automation	-0.58	0.04	-13.19	< 0.001	-0.67, -0.50
Chemistry vs. Automation	-0.66	0.04	-15.04	< 0.001	-0.75, -0.57
Mathematics vs. Automation	-0.97	0.04	-21.90	< 0.001	-1.05, -0.88
Medicine vs. Automation	-0.44	0.04	-9.90	< 0.001	-0.52, -0.35

Note. Beta coefficients represent the mean difference between ratings of automation vs. other disciplines on 1-7 scales, which we describe in the Methods.

In sum, religious people perceived religion as less compatible with automation than with science. These perceptions were partly explained by the view that automation is less associated with laws of nature and that it encourages people to play God. This study supports our finding from Studies S2a-b that religious people feel more negative towards automation than other scientific disciplines, partly because automation gives people capacities that have historically been unique to God.

10. Study S4

Study S4 explored a different mechanism by which automation could lead to religious decline. In our introduction, we focus on how people may see automation as filling the same functional niche as supernatural agents. But it is possible that automation does not lead to religious decline because of functional overlap between automation agents and gods, but simply because of the lifestyles, activities, and challenges that are inherent to working in AI and robotics occupations. Working in AI and robotics may involve challenges that require more concrete (vs. abstract) construal, and lead people to reflect less on their religious values and supernatural beliefs compared to working in other scientific disciplines. We tested this hypothesis with a correlational study.

We ran a pre-registered study in which we asked 196 participants from Amazon Mechanical Turk to rate whether 21 activities were characteristic of AI, medicine (a science control), and telecommunications technology (a technology control). Participants responded to the prompt, “consider the functions of _______. When you use ________, what kinds of challenges are you most frequently trying to solve” using a 1 (“Very Rarely”) – 7 (“Very Frequently”). We also asked a separate sample of 199 religious participants from Amazon Mechanical Turk to rate the same activities using the prompt “What kinds of challenges lead you to feel that religion is important in your life” using a 1 (“Not at All”) – 7 (“Very Much”) scale. The challenges in this study are listed in Table S27.

Table S27.
Items in Study S4

1. Providing connection to a community
2. Providing social support
3. Providing affiliation with others
4. Answering questions about right and wrong
5. Helping to navigate moral issues
6. Trying to live an ethical life
7. Making sense of the world
8. Explaining things that are hard to understand

9. Predicting things that will happen in the near future
10. Solving logistical problems
11. Helping with daily tasks
12. Assisting with your work
13. Making you feel better
14. Providing a sense of comfort
15. Making life easier
16. Helping you discover new things
17. Teaching you about the world
18. Assisting you with learning
19. Performing manual labor
20. Storing things in memory
21. Problem-solving

Challenges associated with AI were negatively associated with likelihood of inspiring religious devotion (henceforth called "religious importance"). For example, challenges such as "answering questions about right and wrong" (M_{AI} = 3.27, M_{REL} = 5.04), and "making sense of the world" (M_{AI} = 3.67, M_{REL} = 5.29) had among the strongest associations with religious importance and the least strong associations with AI. In contrast, "making life easier" (M_{AI} = 3.56, M_{REL} = 4.66), and "assisting with your work" (M_{AI} = 3.27, M_{REL} = 3.65) had among the strongest associations with AI and the least strong associations with religious importance. Whereas association with AI was negatively correlated with religious importance, r = -.51, p = .02, association with telecommunications technology was not significantly correlated with religious importance, r = -.05, p = .83, and association with medicine was positively correlated with religious importance, r = .43, p = .05. These associations are displayed in Figure S7.

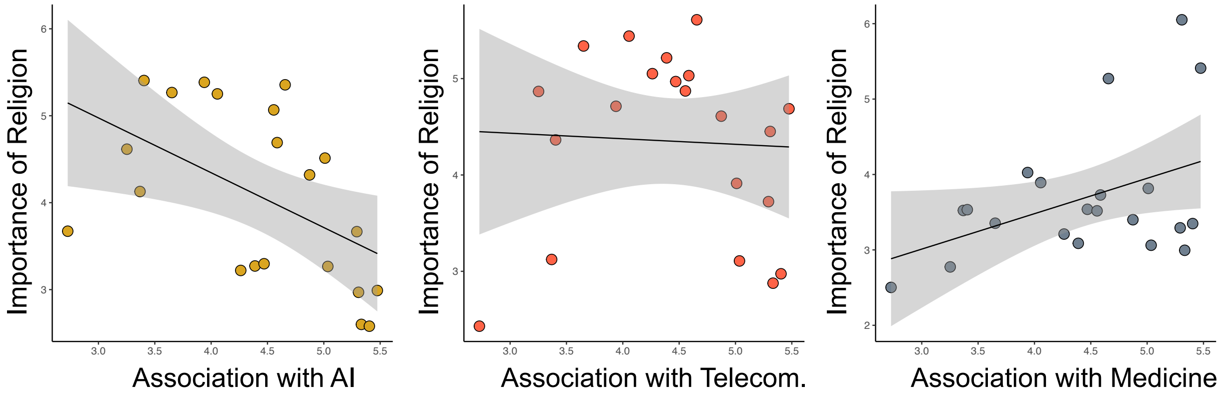

Figure S7. Nodes represent challenges, and the trendline represents the relationship between these challenges' associations with different disciplines and their likelihood of inspiring religious devotion. The error shading represents standard error around these relationships.

These findings suggest that the problems people face when working with automated agents may be uniquely unlikely to inspire religious devotion or strengthen people's religious conviction. This may be one reason why Studies 3-4 (main text) found that exposure to AI professions is associated with more religious decline than exposure to other scientific disciplines.

11. Study S5

Our main text shows that entering a profession in AI is associated with greater religious decline than entering a profession in another scientific field like medicine. In a final pre-registered supplemental study, we tested whether participants could anticipate this religious decline if religious individuals imagined entering an AI-focused occupation vs. an occupation in medicine.

We recruited 402 religiously identified participants for this study using Amazon Mechanical Turk through the CloudResearch platform. Participants in this study were told to imagine that they were accepting a job either in AI or in medicine, with the following prompts:

> ***AI Condition.*** We would like you to imagine that accepting a new job in **computer science** where you frequently use **artificial intelligence**. What kinds of activities do you think you would engage in as part of this job?
>
> ***Medicine Condition.*** We would like you to imagine that accepting a new job in **medicine** where you frequently use **tools of modern medicine**. What kinds of activities do you think you would engage in as part of this job?

Participants were then told that they would be involved in three different activities as part of this new job: (1) diagnosing illnesses faster, (2) developing new medication, and (3) creating treatment plans. We selected these activities because they are obviously in the domain of medicine, but they also have experienced high levels of AI infiltration in recent years.

For each activity, participants responded to the item "using AI [medicine] to solve this problem would strengthen my religious conviction" suing a 1 ("Would Not Strengthen") – 9 ("Would Strengthen") scale. After rating the individual activities, participants also responded to the general item: "As part of your job in _____ [AI/Medicine], how important of a role do you think that religion would play in your life" using a 1 ("Not Important") to 7 ("Very Important") scale.

Participants in the AI condition anticipated less religious conviction when evaluating disease diagnosis, $b = -1.15$, $SE = 0.27$, $t = -4.23$, $p < 0.001$, medicine development, $b = -1.33$, $SE = 0.27$, $t = -5.04$, $p < 0.001$, and treatment plan creation, $b = -1.11$, $SE = 0.27$, $t = -4.17$, $p < 0.001$. Participants also anticipated that they would be less religious upon entering AI vs. medicine, $b = -0.67$, $SE = 0.25$, $t = -2.70$, $p = 0.007$.

This study therefore provides evidence that participants are aware that entering an occupation involving automation might prompt less religiosity. Could this mean that our prior studies involved selection effects, such that non-religious people were simply selecting into AI professions? We find this possibility plausible as a partial explanation of our results, but not a complete explanation. In Study 3, change in religiosity happened within individual over time: working in AI was associated with decreased religiosity over time in the same people. Similarly, participants in Study 4 did not select into working with AI: they were assigned more AI work as their organization integrated AI technology.

References

1. R. Boyd, P. J. Richerson, Culture and the evolutionary process. (University of Chicago press, 1988).

2. Norenzayan, W. M. Gervais, The origins of religious disbelief. Trends. Cogn. Sci. **17**, 20–25 (2013).

3. W. M. Gervais, A. Norenzayan, Analytic thinking promotes religious disbelief. Science. **336**, 493–496 (2012).

4. Shenhav, D. G. Rand, J. D. Greene, Divine intuition: cognitive style influences belief in God. J. Exp. Psychol. Gen. **141**, 423 (2012).

5. Norenzayan, W. M. Gervais, K. H. Trzesniewski, Mentalizing Deficits Constrain Belief in a Personal God. PloS One. **7**, e36880 (2012).

6. J. Jong, J. Halberstadt, Death anxiety and religious belief: An existential psychology of religion. (Bloomsbury Publishing, 2018).

7. W. M. Gervais, M. B. Najle, N. Caluori, The Origins of Religious Disbelief: A Dual Inheritance Approach. Soc. Psychol. Personal. Sci. **12**, 1369–1379 (2021).

8. A. Willard, L. Cingl, Testing theories of secularization and religious belief in the Czech Republic and Slovakia. Evol. Hum. Behav. **38**, 604–615 (2017).

9. A. Willard, L. Cingl, A. Norenzayan, Cognitive biases and religious belief: A path model replication in the Czech Republic and Slovakia with a focus on anthropomorphism. Soc. Psychol. Personal. Sci. **11**, 97–106 (2020).

10. J. Langston, D. Speed, T. J. Coleman III, Predicting age of atheism: credibility enhancing displays and religious importance, choice, and conflict in family of upbringing. Relig. Brain. Behav. **10**, 49–67 (2020).

11. J. A. Lanman, M. D. Buhrmester, Religious actions speak louder than words: exposure to credibility-enhancing displays predicts theism. Relig. Brain. Behav. **7**, 3–16 (2017).

12. J. Henrich, The evolution of costly displays, cooperation and religion: Credibility enhancing displays and their implications for cultural evolution. Evol. Hum. Behav. **30**, 244–260 (2009).

13. S. Atran, Folk biology and the anthropology of science: Cognitive universals and cultural particulars. Behav. Brain. Sci. **21**, 547–569 (1998).

14. P. Boyer, Religion explained: The evolutionary origins of religious thought. (Basic books, 2007).

15. J. L. Barrett, Why would anyone believe in God? (AltaMira Press, 2004).

16. B. G. Purzycki, A. K. Willard, MCI theory: A critical discussion. Relig. Brain. Behav. **6**, 207–248 (2016).

17. W. M. Gervais, J. Henrich, The Zeus problem: Why representational content biases cannot explain faith in gods. J. Cogn. Cult. **10**, 383–389 (2010).

18. T. Swan, J. Halberstadt, The Mickey Mouse problem: Distinguishing religious and fictional counterintuitive agents. PloS One. **14**, e0220886 (2019).

19. B. G. Purzycki, R. A. McNamara, An ecological theory of gods' minds. Cogn. Sci. Relig. Its Philos Implic., pp. 143–167 (2016).

20. N. Epley, B. A. Converse, A. Delbosc, G. A. Monteleone, J. T. Cacioppo, Believers' estimates of God's beliefs are more egocentric than estimates of other people's beliefs. Proc. Natl. Acad. Sci. **106**, 21533–21538 (2009).

21. B. G. Purzycki, The minds of gods: A comparative study of supernatural agency. Cognition. **129**, 163–179 (2013).

22. D. M. Wegner, K. Gray, The mind club: Who thinks, what feels, and why it matters. (Penguin, 2017).

23. N. Spatola, K. Urbanska, God-like robots: the semantic overlap between representation of divine and artificial entities. AI Soc. **35**, 329–341 (2020).

24. C. G. Sibley, J. Bulbulia, Faith after an Earthquake: A Longitudinal Study of Religion and Perceived Health before and after the 2011 Christchurch New Zealand Earthquake. PloS One. **7**, e49648 (2012).

25. J. D. Aten et al., The psychological study of religion and spirituality in a disaster context: A systematic review. Psychol. Trauma. Theory. Res. Pract. Policy. **11**, 597 (2019).

26. N. Caluori, J. C. Jackson, K. Gray, M. Gelfand, Conflict changes how people view God. Psychol. Sci. **31**, 280–292 (2020).

27. J. Henrich et al., War increases religiosity. Nat. Hum. Behav. **3**, 129–135 (2019).

28. P. Norris, R. Inglehart, Sacred and secular: Religion and politics worldwide. (Cambridge University Press, 2011).

29. Grossmann, M. E. Varnum, Social structure, infectious diseases, disasters, secularism, and cultural change in America. Psychol. Sci. **26**, 311–324 (2015).

30. D. J. Bem, A. Allen, On predicting some of the people some of the time: The search for cross-situational consistencies in behavior. Psychol. Rev. **81**, 506 (1974).

31. E. T. Higgins, W. S. Rholes, "Saying is believing": Effects of message modification on memory and liking for the person described. J. Exp. Soc. Psychol. **14**, 363–378 (1978).

32. H. Turpin, Unholy Catholic Ireland: Religious hypocrisy, secular morality, and Irish irreligion. (Stanford University Press, 2022).

33. H. Turpin, M. Andersen, J. A. Lanman, CREDs, CRUDs, and Catholic scandals: Experimentally examining the effects of religious paragon behavior on co-religionist belief. Relig. Brain. Behav. **9**, 143–155 (2019).

34. J. Bentzen, In crisis, we pray: Religiosity and the COVID-19 pandemic. J. Econ. Behav. Org. **192,** 541-583 (2020).

35. F. Shariff, A. K. Willard, T. Andersen, A. Norenzayan, Religious priming: A meta-analysis with a focus on prosociality. Personal. Soc. Psychol. Rev. **20**, 27–48 (2016).

36. Norenzayan, A. F. Shariff, The origin and evolution of religious prosociality. Science, **322**, 58–62 (2008).

37. M. E. Aveyard, A call to honesty: Extending religious priming of moral behavior to Middle Eastern Muslims. PloS One. **9**, e99447 (2014).

38. Randolph-Seng, M. E. Nielsen, Honesty: One effect of primed religious representations. Int. J. Psychol. Relig. **17**, 303–315 (2007).

39. R. Sosis, Does religion promote trust?: The role of signaling, reputation, and punishment. Interdiscip. J. Res. Relig. **1** (2005).

40. L. Hall, A. B. Cohen, K. K. Meyer, A. H. Varley, G. A. Brewer, Costly signaling increases trust, even across religious affiliations. Psychol. Sci. **26**, 1368–1376 (2015).

41. M. E. McCullough, E. C. Carter, “Religion, self-control, and self-regulation: How and why are they related?” in APA handbook of psychology, religion, and spirituality (Vol 1): Context, theory, and research, K. I. Pargament, J. J. Exline, W. Jones, Eds. (American Psychological Association, 2013), pp. 123 - 138.

42. M. E. McCullough, B. L. Willoughby, Religion, self-regulation, and self-control: Associations, explanations, and implications. Psychol. Bull. **135**, 69 (2009).

43. J. Jackson, J. Halberstadt, J. Jong, H. Felman, Perceived openness to experience accounts for religious homogamy. Soc. Psychol. Personal. Sci. **6**, 630–638 (2015).

44. H. F. Lyle, E. A. Smith, The reputational and social network benefits of prosociality in an Andean community. Proc. Natl. Acad. Sci. **111**, 4820–4825 (2014).

45. D. T. De Ridder, G. Lensvelt-Mulders, C. Finkenauer, F. M. Stok, R. F. Baumeister, Taking stock of self-control: A meta-analysis of how trait self-control relates to a wide range of behaviors. Personal. Soc. Psychol. Rev. **16**, 76–99 (2012).

46. N. K. Malhotra, S. S. Kim, A. Patil, Common method variance in IS research: A comparison of alternative approaches and a reanalysis of past research. Manag. Sci. **52**, 1865–1883 (2006).

47. C. R. Wanberg, J. Zhu, E. A. Van Hooft, The job search grind: Perceived progress, self-reactions, and self-regulation of search effort. Acad. Manage. J. **53**, 788–807 (2010).

48. M. S. Mitchell, R. L. Greenbaum, R. M. Vogel, M. B. Mawritz, D. J. Keating, Can you handle the pressure? The effect of performance pressure on stress appraisals, self-regulation, and behavior. Acad. Manage. J. **62**, 531–552 (2019).

49. K. Lee, N. J. Allen, Organizational citizenship behavior and workplace deviance: the role of affect and cognitions. J. Appl. Psychol. **87**, 131 (2002).

50. R. J. Bennett, S. L. Robinson, Development of a measure of workplace deviance. J. Appl. Psychol. **85**, 349 (2000).

51. J. Koopman, S. H. Lin, A. C. Lennard, F. K. Matta, R. E. Johnson, My coworkers are treated more fairly than me! A self-regulatory perspective on justice social comparisons. Acad. Manage. J. **63**, 857–80 (2020).

52. D. Welsh, J. Bush, C. J. Thiel, J. Bonner, Reconceptualizing goal setting’s dark side: The ethical consequences of learning versus outcome goals. Organ. Behav. Hum. Decis. Process. **150**, 14–27 (2019).

53. S. L. Robinson, Trust and breach of the psychological contract. Adm. Sci. Q. **41,** 574–99 (1996).

54. W. M. Gervais, A. Norenzayan, Like a camera in the sky? Thinking about God increases public self-awareness and socially desirable responding. J. Exp. Soc. Psychol. **48**, 298–302 (2012).

55. R. Sosis, B. J. Ruffle, Religious ritual and cooperation: Testing for a relationship on Israeli religious and secular kibbutzim. Curr. Anthropol. **44**, 713–22 (2003).

56. C. Leicht, C.A. Sharp, J. P. LaBouff, N. Zarzeczna, F. Elsdon-Baker, Content matters: Perceptions of the science-religion relationship. Int. J. Psychol. Relig. **32**, 232–55 (2022).

57. E. H. Ecklund, Science vs. religion: What scientists really think. (Oxford University Press, 2010).

58. T. Davoodi, T. Lombrozo, Explaining the existential: scientific and religious explanations play different psychological roles, J. Exp. Psych. Gen. **151,** 1199-1218 (2022).

59. J. C. Jackson, D. Dillion, B. Bastian, J. Watts, N. DiMaggio, K. Gray (2021) Supernatural explanations across 114 societies are more common for Natural than social phenomena, Nat. Hum. Beh. **7**, 707-717 (2023).

60. G. Inc Religion. Gallup.com. Available at: https://news.gallup.com/poll/1690/Religion.aspx (Accessed: December 28, 2021).

61. W. M. Gervais, M. B. Najle, How many atheists are there? Soc. Psychol. Personal. Sci. **9**, 3–10 (2018).

62. Anonymous, Communist Countries, Past and Present. Infoplease.com. Available at: https://www.infoplease.com/world/diplomacy/communist-countries-past-and-present (Accessed: February 13, 2022).
